# Supplementary material for: People’s Values and Preferences about Meat Consumption in View of the Potential Environmental Impacts of Meat: A Mixed-methods Systematic Review
Source: Int J Environ Res Public Health. 2022 Dec 24;20(1):286. doi: 10.3390/ijerph20010286 (PMC9819158; doi:10.3390/ijerph20010286)
Supplement: Supplementary file 1 [file ijerph-20-00286-s001.zip › ijerph-2052270-supplementary.pdf]

Supplementary Material

Contents

Supplementary Material 1

Supplement Table 1. Search Strategies 2

Supplement Table 2. Quantitative Data Extraction Form 4

Supplement Table 3. Qualitative Data Extraction Form 5

Supplement Table 4. Critical Meta-narrative Synthesis: From Quantitative Data to Narratives 7

Supplement Table 5. Excluded Studies and Reasons for Exclusion (N=357) 8

Supplement Table 6. Risk-of-Bias Assessments for Quantitative Studies 39

Supplement Table 7. Methodological Limitations Assessments for Qualitative Studies 41

Supplement Table 8. Critical appraisal for Mixed-Methods studies 43

Supplement Table 9. Evidence Profile for “Reasons for eating and/or buying meat” 44

Supplement Table 10. Evidence Profile for “Reasons for avoiding meat” 45

Supplement Table 11. Evidence Profile for “Willingness to Change Meat Consumption” 47

Supplement Table 12. Evidence Profile for “Willingness to pay more for environmentally friendly meat” 49

Supplement Table 1. Search Strategies

MEDLINE<br>PubMed<br>19/10/2020	((("Food Preferences"[MeSH Terms] OR "preference*"[Title/Abstract] OR "Consumer Behavior"[MeSH Terms] OR "choice*"[Title/Abstract] OR "view*"[Title] OR "expectation*"[Title] OR "attitude*"[Title/Abstract] OR "knowledge*"[Title] OR "belief*"[Title] OR "accepta*"[Title] OR "perception*"[Title] OR "opinion*"[Title] OR "perspective*"[Title] OR "consumer*"[Title] OR "Diet surveys"[MeSH Terms]) AND ("Meat"[MeSH Terms] OR "Meat"[Title] OR "red meat"[Title/Abstract] OR "processed meat"[Title/Abstract] OR "meat product*"[Title/Abstract] OR "sausage*"[Title/Abstract] OR "ham"[Title/Abstract] OR "hams"[Title/Abstract] OR "bacon"[Title/Abstract] OR "salami"[Title/Abstract] OR (("Meat"[Title/Abstract] OR "meats"[Title/Abstract] OR "Food"[MeSH Terms] OR "Feeding Behavior"[MeSH Terms] OR "diet, food, and nutrition"[MeSH Terms]) AND ("beef"[Title/Abstract] OR "mutton"[Title/Abstract] OR "horse"[Title/Abstract] OR "bovine"[Title/Abstract] OR "veal"[Title/Abstract] OR "lamb"[Title/Abstract] OR "goat"[Title/Abstract] OR "pork"[Title/Abstract]))) **5826**
Ovid Embase <1974 to 2019 Week 23><br>01/08/2020	1 exp food preference/<br>2 exp consumer attitude/<br>3 preference*.ti,ab.<br>4 choice*.ti,ab.<br>5 view*.ti.<br>6 expectation*.ti.<br>7 attitude*.ti,ab.<br>8 knowledge*.ti.<br>9 belief*.ti.<br>10 accepta*.ti.<br>11 perception*.ti.<br>12 opinion*.ti.<br>13 perspective*.ti.<br>14 consumer*.ti.<br>15 1 or 2 or 3 or 4 or 5 or 6 or 7 or 8 or 9 or 10 or 11 or 12 or 13 or 14<br>16 exp meat/<br>17 meat.ti.<br>18 red meat.ti,ab.<br>19 processed meat.ti,ab.<br>20 meat product*.ti,ab.<br>21 sausage*.ti,ab.<br>22 ham.ti,ab.<br>23 hams.ti,ab.<br>24 bacon.ti,ab.<br>25 salami.ti,ab.)<br>26 16 or 17 or 18 or 19 or 20 or 21 or 22 or 23 or 24 or 25<br>27 meat.ti,ab.<br>28 meats.ti,ab.<br>29 exp food/<br>30 exp feeding behavior/<br>31 27 or 28 or 29 or 30<br>32 beef.ti,ab.<br>33 mutton.ti,ab.<br>34 horse.ti,ab.<br>35 bovine.ti,ab.<br>36 veal.ti,ab.<br>37 lamb.ti,ab.<br>38 goat.ti,ab.<br>39 duck.ti,ab.<br>40 pork.ti,ab.<br>41 chicken*.ti,ab.<br>42 poultry.ti,ab.<br>43 turkey.ti,ab.<br>44 hen.ti,ab.<br>45 goose.ti,ab.

	46 rabbit.ti,ab.<br>47 32 or 33 or 34 or 35 or 36 or 37 or 38 or 39 or 40 or 41 or 42 or 43 or 44 or 45 or 46<br>48 31 and 47<br>49 26 or 48<br>50 15 and 49 **5733**
AGRIS<br>12/10/2020	(preference OR (“consumer behavior” OR (choice OR (view OR (expectation OR (attitude OR (knowledge OR (belief OR (accepta* OR (perception OR (opinion OR (perspective OR consumer))))))))))) AND ((meat OR (processed OR (sausage OR (ham OR (bacon OR salami))))) OR ((meat OR (food OR (beef OR (mutton OR (horse OR (bovine OR (veal OR(lamb OR(goat OR pork)))))))) AND (meat OR (food OR ("feeding behaviour"))))) AND adults **1192**
FSTA<br>09/10/2020	S1 DE "CONSUMER RESPONSE" 41,38<br>S2 TX preference* OR choice* OR view* OR expectation* OR attitude OR knowledge OR belief* OR accepta* OR perception* OR opinion* OR consumer* 176,759<br>S3 S1 OR S2 176,759<br>S4 DE “meat specific” 10,135<br>S5 TX meat* OR sausage* OR ham* OR bacon OR salami OR beef OR mutton OR horse OR bovine OR veal OR lamb OR goat OR pork OR duck OR chicken OR poultry OR hen OR goose OR rabbit 238,946<br>S6 S4 OR S5 238,946<br>S7 DE "nutrition" Expanders 27,328<br>S8 TX diet OR nutrition* 289,898<br>S9 S7 OR S8 289,898<br>S10 S3 AND S6 AND S9 **7858**
CAB Abstracts (CAB Direct)<br>09/10/2020	((ab:("red meat" OR "processed meat") OR title:("red meat" OR "processed meat" OR sausage* OR ham OR hams OR bacon OR salami OR meat) OR "meat") OR (("eating behaviour" OR title:(meat OR meats)) AND (title:(beef OR mutton OR horse OR bovine OR veal OR lamb OR goat OR duck OR pork OR chicken* OR poultry OR turkey OR hen OR goose OR rabbit)))) AND (title:(preference* OR choice* OR attitude* OR view* OR expectation* OR knowledge* OR belief* OR accepta* OR perception* OR opinion* OR perspective* OR consumer*) OR ("feeding preferences" OR "consumer attitudes" OR "consumer behaviour")) **6938**
Web of science<br>10/08/2020	(TS= (Food Preferences)) OR (TI= (preference*)) OR (TS= (Consumer Behavior)) OR (TI= (choice*) OR (view*) OR (expectation*) OR (attitude*) OR (knowledge*) OR (belief*) OR (accepta*) OR (perception*) OR (opinion*) OR (perspective*) OR (consumer*)) OR (TS=(Diet surveys)) AND (TS= (Meat)) OR (TI= (meat) OR (red meat) OR (processed meat) OR (meat product*) OR (sausage*) OR (ham) OR (hams) OR (bacon) OR (salami)) OR (TI=(meat) OR (meats) OR (TS=(Food) OR (Feeding Behavior) OR (Diet, Food, and Nutrition)) AND (TI=(beef) OR (mutton) OR (horse) OR (bovine) OR (veal) OR (lamb) OR (goat) OR (duck) OR (pork) OR (chicken*) OR (poultry) OR (turkey) OR (hen) OR (goose) OR (rabbit)) **3171**

Supplement Table 2. Quantitative Data Extraction Form

**Variable**
**Researcher identification**
1. Surname, name
2. Date
**Study identification**
3. Id
4. Organization
5. Country
6. Conflict of interest
**Study objectives or research questions**
7. Objectives or research questions
**Study population**
8. Study setting
9. Participants socio-demographic characteristics
10. Participants health status
11. Participants meat consumption behaviour
12. Sample size
13. Sampling strategy
14. Recruitment process
**Study design and methods**
15. Study design
16. Study period
17. Choice of measurement instrument
**Risk of Bias**
18. Selection of participants (Risk of bias 1)<br>Was an appropriate study sample selected from the sampling frame? (Consider: is the study sample representative for the target population? Choose the option you think is the most appropriate one for this question)
19. Missing data (Risk of bias 2)<br>Was the response rate sufficiently high to minimize the risk of bias? (To consider: was the response rate ⩾ 60 %?)
20. Measurement instrument (Risk of bias 3)<br>Did the researchers pilot the measurement techniques on a subset of the target population? Was the instrument validated? Was the instrument reliable?
**Findings**
21. Main findings
22. Authors' interpretation
23. Authors' conclusions

Supplement Table 3. Qualitative Data Extraction Form

Variable
**Researcher identification**
1. Surname, name
2. Date
**Study identification**
3. Id
4. Organization
5. Country
6. Conflict of interest
7. Qualitative approach
**Study population**
8. Study setting
9. Participants socio-demographic characteristics
10. Participants health status
11. Participants meat consumption behaviour
12. Sample size
**Risk of Bias**
13. CASP checklist question 1<br>Was there a clear statement of the aims of the research?<br>HINT: Consider<br>• What was the goal of the research?<br>• Why it was thought important?<br>Its relevance<br>Please consider the goal of the research and whether it is clearly defined.
14. CASP checklist question 2<br>Is a qualitative methodology appropriate?<br>HINT: Consider<br>If the research seeks to interpret or illuminate the actions and/or subjective experiences of research participants<br>Is qualitative research the right methodology for addressing the research goal?
15. CASP checklist question 3<br>Was the research design appropriate to address the aims of the research?<br>HINT: Consider<br>If the researcher has justified the research design (e.g., have they discussed how they decided which method to use)?
16. CASP checklist question 4<br>Was the recruitment strategy appropriate to the aims of the research?<br>HINT: Consider<br>If the researcher has explained how the participants were selected<br>• If they explained why the participants they selected were the most appropriate to provide access to the type of knowledge sought by the study.<br>If there are any discussions around recruitment (e.g. why some people chose not to take part)<br>Please also consider:<br>• Who was included in the study?<br>• Who was excluded from the study?<br>Is the sample appropriate in terms of its ability to meet the aims of the study, the depth of data that it is enables to be collected, and its breadth?<br>• How was the sample selected? Were there any factors that influenced how the sample was selected (e.g. access, timescale issues)?
17. CASP checklist question 5<br>Was the data collected in a way that addressed the research issue?<br>HINT: Consider<br>If the setting for data collection was justified<br>• If it is clear how data were collected (e.g. focus group, semi-structured interview etc.)<br>• If the researcher has justified the methods chosen<br>• If the researcher has made the methods explicit (e.g. for interview method, is there an indication of how interviews were conducted, or did they use a topic guide)?

• If methods were modified during the study. If so, has the researcher explained how and why?<br>• If the form of data is clear (e.g. tape recordings, video material, notes etc.)<br>If the researcher has discussed saturation of data<br>Inappropriate methods, such as closed questions in interviews and surveys; inappropriate combination of qualitative design with quantitative design<br>• What data collection methods were used?<br>• Over what period did the data collection take place?
18. <u>CASP checklist question 6</u><br>Has the relationship between researcher and participants been adequately considered?<br>HINT: Consider<br>• If the researcher critically examined their own role, potential bias and influence during<br>(a) Formulation of the research questions<br>(b) Data collection, including sample recruitment and choice of location<br>How the researcher responded to events during the study and whether they considered the implications of any changes in the research design<br>Please also consider:<br>Are the researcher's /researchers' own position, assumptions and possible biases outlined? If any, indicate how they could affect the study in terms of analysis and interpretation of the data
19. <u>CASP checklist question 7</u><br>Have ethical issues been taken into consideration?<br>HINT: Consider<br>• If there are sufficient details of how the research was explained to participants for the reader to assess whether ethical standards were maintained<br>• If the researcher has discussed issues raised by the study (e.g. issues around informed consent or confidentiality or how they have handled the effects of the study on the participants during and after the study)<br>If approval has been sought from the ethics committee<br>Please also consider:<br>• Was ethical committee approval obtained?<br>• Was informed consent obtained?<br>• Does the study address ethical issues adequately?<br>• Has confidentiality been maintained?
20. <u>CASP checklist question 8</u><br>Was the data analysis sufficiently rigorous?<br>HINT: Consider<br>• If there is an in-depth description of the analysis process<br>• If thematic analysis is used. If so, is it clear how the categories/themes were derived from the data?<br>• Whether the researcher explains how the data presented were selected from the original sample to demonstrate the analysis process<br>• If sufficient data are presented to support the findings<br>• To what extent contradictory data are taken into account<br>Whether the researcher critically examined their own role, potential bias and influence during analysis and selection of data for presentation<br>Please also consider (you don't have to answer the questions one-by-one):<br>• How are the data analysed?<br>• How adequate is the description of the data analysis?<br>• Is adequate evidence provided to support the analysis (e.g. use of original data, iterative analysis, efforts to establish validity and reliability)?<br>• Is the study set in context in terms of findings and relevant theory?<br>Are the findings substantiated by the data and has consideration been given to any limitations of the methods or data that may have affected the results?
21. <u>CASP checklist question 9</u><br>Is there a clear statement of findings?<br>HINT: Consider<br>• If the findings are explicit<br>• If there is adequate discussion of the evidence both for and against the researchers' arguments<br>• If the researcher has discussed the credibility of their findings (e.g. triangulation, respondent validation, more than one analyst)

If the findings are discussed in relation to the original research question
22. CASP checklist question 10<br>How valuable is the research?<br>HINT: Consider<br>• If the researcher discusses the contribution the study makes to existing knowledge or understanding e.g. Do they consider the findings in relation to current practice or policy? Or relevant research-based literature?<br>• If they identify new areas where research is necessary<br>If the researchers have discussed whether or how the findings can be transferred to other populations or considered other ways the research may be used
**Findings**
24. Main findings
25. What are the themes/codes/topics?
26. Authors' interpretation
27. Authors' conclusions

Supplement Table 4. Critical Meta-narrative Synthesis: From Quantitative Data to Narratives

Systematic profiles			Critical questions
Technique	Focus	Example	• What is the study trying to say about participants values?<br>• Are participants' values explicitly identified? If so, what are they?<br>• How do participants' answers to the question on their willingness to change/reduce meat consumption?<br>• Are there other individual or contextual factors (such as age, gender, education, socioeconomic status) that influence participants' values?<br>• How different are participants'' perspective on meat consumption?
Modal profile	The most and different occurring attributes	If the majority of study participants reported to consume meat, they were described as omnivores.	
Average profile	Average of a particular variable	"Almost 50% of the respondents agree, and 24% disagree, that it is a good idea for the environment to have a meat-free day per week. In comparison, only 14% of the respondents claim to actually have reduced their consumption of meat due to environmental reasons and 34% of the respondents find it hard to reduce their meat consumption". This finding was transformed as follows: "*Although participants agreed/believed that a reduction of meat intake would benefit the environment, most of the participants who reported to have reduced their intake in the past did not do it for environmental reasons.*"	
Comparative profile	A comparison of key outcomes	Participants were asked to ranked the importance of different aspects when buying meat. The higher the mean score, the greater the importance. Ratings were made on a seven-point scale anchored by "Not important at all" to "Extremely important"):<br>1. Low price 4.32 (0.78)<br>2. Freshness 5.57 (0.79)<br>3. Food Safety 5.61 (0.76)<br>4. No medicine residues 5.39 (0.79)<br>5. Environmentally friendly production 4.76 (1.00)<br>6. Taste 5.52 (1.60)<br>7. Animal welfare orientated production 4.87 (1.18)<br>8. Natural production 4.73 (1.16)<br>9. Healthy product 5.55 (1.17)<br>This was transformed as follows: "*When participants were asked to report which meat attribute was important when buying/consuming meat, the environment was not considered the most important characteristic, other aspects were considered as important or more important.*"	
Holistic profile	A combination of the modal, average and comparative profiles	"Regarding the statement: ´To help reduce the impact of climate change, it is better to eat less animal foods (meat, dairy products and eggs) ´:<br>- No significant difference in degree of agreement on the statement was found between male and female respondents ($\chi2$ =3.21; P≥0·05)<br>- No significant difference in degree of agreement on the statement was found between age groups ($\chi2$ =7.86; P≥0·05)<br>- No significant different in degree of agreement on the statement was found between socio-economic groups ($\chi2$ =2.85; P≥0·05)<br>- No significant difference in degree of agreement on the statement was found between low meat eaters (≤1 portion of meat/d) and high meat eaters (>1 portion of meat/d) ($\chi2$ =1.29; P≥0·05))". It was transformed as follows: *Socio-economic status, meat consumption behaviour and gender were not associated*	

		with peoples' belief that eating less meat would reduce the influence climate change	

Supplement Table 5. Excluded Studies and Reasons for Exclusion (N=359)

**Study ID**	**Reason**
Aker 2004	Conference abstract
Antunes 2016	Conference abstract
Gardini 2015	Conference abstract
Jensen 2012	Conference abstract
Lawrence 2017	Conference abstract
Randel 2014	Conference abstract
Resano 2010	Conference abstract
Richmond 2015	Conference abstract
Sanderson 2015	Conference abstract
Thaxton 2016	Conference abstract
Touillaud 2012	Conference abstract
ICoMST 2009	Editorial
Joo 2010	Editorial
Kowalkowska 2015	Exclusion criteria (at least 30% teenagers)
Gorecka 2009	Exclusion criteria (functional food and content of fat in meat)
Mauracher 2013	Exclusion criteria (meat alternatives)
Napolitano 2010	Exclusion criteria (meat alternatives)
Allen 2003	Exclusion criteria (meat composition)
Flight 2003	Exclusion criteria (meat composition)
Pourová 2002	Exclusion criteria (meat composition)
Hartmann 2015	Exclusion criteria (meat quality)
Wilmer 2010	Exclusion criteria (meat quality)
Nantes 2014	Exclusion criteria (Outside EU/USA/Australia/Canada: study from Brazil)
Schnettler 2008	Exclusion criteria (Outside EU/USA/Australia/Canada: study from Chile)
Hong 2018	Exclusion criteria (Outside EU/USA/Australia/Canada: study from China)
Xu 2019	Exclusion criteria (Outside EU/USA/Australia/Canada: study from China)
Yin 2020	Exclusion criteria (Outside EU/USA/Australia/Canada: study from China)
Lai 2017	Exclusion criteria (Outside EU/USA/Australia/Canada: study from China)
Sonoda 2018	Exclusion criteria (Outside EU/USA/Australia/Canada: study from Japan)
Zlaoui 2014	Exclusion criteria (Outside EU/USA/Australia/Canada: study from Tunisia)

Arenas de Moreno 2020	Exclusion criteria (Outside EU/USA/Australia/Canada: study from Venezuela)
Munni 2017	Exclusion criteria (Outside EU/USA/Australia: diabetic pregnant women from Bangladesh)
Davaasuren 2017	Exclusion criteria (Outside EU/USA/Australia: Mongolian population)
Jen 2015	Exclusion criteria (Outside EU/USA/Australia: Taiwanese meat consumers)
Delanoue 2014	Exclusion criteria (safety)
Salminen 2002	Exclusion criteria (Specific population =cancer survivors)
Pribis 2010	Exclusion criteria (Specific population =Seventh day Adventist (SDA) religious group).
Roos 2001	Exclusion criteria (Specific population =variation in occupational groups)
Bovell-Benjamin 2013	Exclusion criteria (Specific population= cancer survivors)
Maskarine 2001	Exclusion criteria (Specific population= cancer survivors)
Bonne 2006	Exclusion criteria (Specific population=Muslim/halal meat).
Elmubarak 2004	Exclusion criteria (Specific population=Sudanese Americans).
Hu 2003	Exclusion criteria (Specific population=Sudanese Americans).
Bohm 2015	Exclusion criteria (teenager/ children)
Firetti 2010	No exposure of interest
Hulya 2011	No exposure of interest
Karakaya 2014	No exposure of interest
McCarthy 2004	No exposure of interest
Protino 2013	No exposure of interest
Santos 2011	No exposure of interest
Altintzoglou 2011	No exposure of interest (fish /seafood)
Clonan 2011	No exposure of interest (fish /seafood)
Hall 2013	No exposure of interest (fish /seafood)
Honkanen 2009_1	No exposure of interest (fish /seafood)
Jaffry 2004	No exposure of interest (fish /seafood)
Morales 2018	No exposure of interest (fish /seafood)
Pieniak 2010_1	No exposure of interest (fish /seafood)
Pieniak 2010_2	No exposure of interest (fish /seafood)
Stefani 2012	No exposure of interest (fish /seafood)
Thong 2016	No exposure of interest (fish /seafood)
vanDijk 2011	No exposure of interest (fish /seafood)
Verbeke 2005	No exposure of interest (fish /seafood)
Verbeke 2007	No exposure of interest (fish /seafood)
Schlup 2018	No exposure of interest (insects)

Verbeke 2014_2	No exposure of interest (insects)
Morales 2018	No exposure of interest (fish /seafood)
Clonan 2011	No exposure of interest (fish /seafood)
Hall 2013	No exposure of interest (fish /seafood)
Jaffry 2013	No exposure of interest (fish /seafood)
Stefani 2012	No exposure of interest (fish /seafood)
Schlup 2018	No exposure of interest (insects)
Emberger-Klein 2018	No exposure of interest (it is about food in general)
Rani 2013	No exposure of interest (mutton meat)
Bryant 2019	No exposure of interest (plant-based meat)
Bierman 2020	No exposure of interest and no findings of interest (food in general and not focused on V&P regarding EC)
Joyce 2008	No exposure of interest (nutritional knowledge)
Lee 2016	No exposure of interest (nutritional knowledge)
Bodnar 2010	No exposure of interest (game meat)
Adamski 2017	No findings of interest
Ali 2017	No findings of interest
Allen 2000	No findings of interest
Altintzoglou 2010a	No findings of interest
Altintzoglou 2010b	No findings of interest
Andrade 2013	No findings of interest
Apostolidis 2016	No findings of interest
Aral 2013	No findings of interest
Arica 2017	No findings of interest
Armağan 2005	No findings of interest
Arthur 2011	No findings of interest
Azevedo 2016	No findings of interest
Backer 2014	No findings of interest
Bailey 2014	No findings of interest
Balcombe 2016	No findings of interest
Ballantyne 2006	No findings of interest
Barrena 2009	No findings of interest
Bell 2017	No findings of interest
Bennett 2002a	No findings of interest
Bennett 2002b	No findings of interest
Bennett 2012	No findings of interest
Bergstra 2017	No findings of interest
Berndsen 2004	No findings of interest
Berndsen 2005	No findings of interest
Blake 2007	No findings of interest
Blasco 2017	No findings of interest
Bouwman 2009	No findings of interest
Bovell-Benjamin 2009	No findings of interest
Bratanova 2011	No findings of interest

Brscic 2013	No findings of interest
Burger 2009	No findings of interest
Butterworth 2005	No findings of interest
Büyükkaragöz 2014	No findings of interest
Caklova 2014	No findings of interest
Campbell 2016	No findings of interest
Caracciolo 2016	No findings of interest
Carlsson 2007	No findings of interest
Cerjak 2011	No findings of interest
Chea 2020	No findings of interest
Christensen 2006	No findings of interest
Clonan 2015	No findings of interest
Corcoran 2001	No findings of interest
Cox 2016	No findings of interest
Dagevos 2014	No findings of interest
DeBarcellos 2011	No findings of interest
deBoer 2016	No findings of interest
DeBoer 2007	No findings of interest
DeHouwer 2007	No findings of interest
Deimel 2010	No findings of interest
deJonge 2013	No findings of interest
deJonge 2014	No findings of interest
delCoz 2006	No findings of interest
Dentoni 2010	No findings of interest
Derell 2016	No findings of interest
Dockès 2011	No findings of interest
Dreezens 2005	No findings of interest
Ebewore 2015	No findings of interest
EFSA 2011	No findings of interest
EFSA 2012	No findings of interest
El-Abbadi 2017	No findings of interest
El-Naga 2015	No findings of interest
EuropeanCommision 2007	No findings of interest
EuropeanCommision2005	No findings of interest
Feeley 2014	No findings of interest
Fishman 2003	No findings of interest
Fotea 2012	No findings of interest
Frewer2005	No findings of interest
Gaspar 2016	No findings of interest
Gittelsohn 2003	No findings of interest
Graca 2015	No findings of interest
Gracia 2013	No findings of interest
Grunert 2003	No findings of interest
Gutkowska 2011	No findings of interest
Hamlin 2016	No findings of interest
Haper 2001	No findings of interest

Hayley 2015	No findings of interest
Heid 2011	No findings of interest
Heleski 2004	No findings of interest
Heleski 2006	No findings of interest
Henchion 2017	No findings of interest
Herzog 2009	No findings of interest
Hilverda 2016	No findings of interest
Hobbs 2005	No findings of interest
Hoek 2010	No findings of interest
Hong 2006	No findings of interest
Hovi 2003	No findings of interest
Hsu 2014	No findings of interest
Hung 2016	No findings of interest
Hunter 2016	No findings of interest
Hygreeva 2014	No findings of interest
Inoue 2006	No findings of interest
Ippolitov 2015	No findings of interest
Izmirli 2010	No findings of interest
Jacobs 2015	No findings of interest
Juma 2010	No findings of interest
Kalof1999	No findings of interest
Kennewell 2001	No findings of interest
Kjaernes 2007	No findings of interest
Klöckner 2016	No findings of interest
Knight 2004	No findings of interest
Koizumi 2000	No findings of interest
Koritar 2017	No findings of interest
Kosicka-Gebska 2013	No findings of interest
Krystallis 2009	No findings of interest
Kulkarni 2009	No findings of interest
Kumar 2014	No findings of interest
Lagerkvist 2006	No findings of interest
Lassen 2016	No findings of interest
Lea 2008	No findings of interest
Lepore 2014	No findings of interest
Liljenstolpe 2008	No findings of interest
Lipoeto 2001	No findings of interest
Lopez Osorino 2007	No findings of interest
Loughnan 2010	No findings of interest
Love 2008	No findings of interest
Mäkiniemi 2014	No findings of interest
Marin 2016	No findings of interest
Matwiejczyk 2016	No findings of interest
Mayfield 2007	No findings of interest
McCarthy 2005	No findings of interest
McIntyre 2009	No findings of interest

McKendree 2014	No findings of interest
Mejia 2012	No findings of interest
Mijatovic 2012	No findings of interest
Miranda-de la Lama 2017	No findings of interest
Mooney 2001	No findinigs of interest
MoraisSato 2014	No findings of interest
Muringai 2016	No findings of interest
Nanni 2007	No findings of interest
Narchi 2008	No findings of interest
Nath 2010	No findings of interest
Neo 2016	No findings of interest
Ngapo 2004	No findings of interest
Nielsen 2009	No findings of interest
O'Rouke 2004	No findings of interest
Oakes 2006	No findings of interest
Ogut 2008	No findings of interest
Olewnik-Mikolajewska 2016	No findings of interest
Oliveros 2012	No findings of interest
Olsen 2008	No findings of interest
Oteku 2006	No findings of interest
Ozawa 2004	No findings of interest
Özen 2009	No findings of interest
Patil 2004	No findings of interest
Paul 2000	No findings of interest
Petroka 2016	No findings of interest
Pfeiler 2017	No findings of interest
Pfeiler 2018	No findings of interest
Phillips 2012	No findings of interest
Piqueras-Fiszman 2016	No findings of interest
Pirsich 2016	No findings of interest
Popescu 2013	No findings of interest
Popluga 2010	No findings of interest
Povey 2001	No findings of interest
Preylo 2008	No findings of interest
Prunty 2013	No findings of interest
Pufpaff 2014	No findings of interest
Qing 2014	No findings of interest
Queiroz 2014	No findings of interest
Radder 2003	No findings of interest
Radman 2005	No findings of interest
Raghavendra 2009	No findings of interest
Raineri 2012	No findings of interest
Rathod 2011	No findings of interest
Rothgerber 2014	No findings of interest
Rothgerber 2015a	No findings of interest
Rothgerber 2015b	No findings of interest

Rousset 2005	No findings of interest
Ruby 2016	No findings of interest
Rurik 2006	No findings of interest
Rutsaert 2015	No findings of interest
Santos 2015	No findings of interest
Santos 2017	No findings of interest
Schaly 2010	No findings of interest
Scott 2007	No findings of interest
Scozzafava 2014	No findings of interest
SegoviaLopez 2007	No findings of interest
Seker 2011	No findings of interest
Serpell 2005	No findings of interest
Shan2017	No findings of interest
Shepherd 2001	No findings of interest
Shipman 2017	No findings of interest
Siegrist 2015	No findings of interest
Sigman-Grant 2008	No findings of interest
Simoes 2015	No findings of interest
Song 2014	No findings of interest
Sow 2010	No findings of interest
Strijbos 2016	No findings of interest
Suresh 2016	No findings of interest
Szendro 2016	No findings of interest
Thorslund 2017	No findings of interest
Tian 2015	No findings of interest
Trocchia 2003	No findings of interest
Tsegay 2012	No findings of interest
Urland 2005	No findings of interest
Van Wezemael 2010	No findings of interest
Van Zyl 2010	No findings of interest
Vanhonacker 2009	No findings of interest
Vanhonacker 2010	No findings of interest
Vanhonacker 2013	No findings of interest
Vartanian 2015	No findings of interest
Velde 2002	No findings of interest
Viana 2014	No findings of interest
Vida 2013	No findings of interest
Vukasovic 2014	No findings of interest
Wang 2014	No findings of interest
Wardy 2014	No findings of interest
Wilson 2002	No findings of interest
Yercan 2014	No findings of interest
Young-Mee 2008	No findings of interest
Zanoli 2011	No findings of interest
Zhang 2001	No findings of interest
Zheng 2016	No findings of interest

Zingg 2012	No findings of interest
Akrofi 2019	No findings of interest
Aschemann-Witzel 2019	No findings of interest
Bert 2019	No findings of interest
Bir 2019	No findings of interest
Bruno 2019	No findings of interest
Chong 2019	No findings of interest
Feinberg 2019	No findings of interest
Kause 2019	No findings of interest
Krispenz2020	No findings of interest
Mertens 2020	No findings of interest
Tarrega 2020	No findings of interest
Willits-Smith 2020	No findings of interest
Shi 2016	No findings of interest
Kurz 2018	No findings of interest
Harper 2001	No findings of interest
Hong 2006	No findings of interest
Harray 2018	No findings of interest
Trevena 2007	No findings of interest
Carroll 2019	No findings of interest
Wang 2019	No findings of interest
Plante 2019	No findings of interest (it is a correlation study)
Kjaerness 2005	No findings of interest (animal welfare)
Rice 2020	No findings of interest (animal welfare) Study design (it is an intervention/experimental study)
De Backer 2020	No findings of interest (masculinity and meat consumption not related to the environment)
Lea 2006	No findings of interest (reasons for eating plants)
Shab 2018.	No findings of interest. Exclusion criteria (Outside EU/USA/Australia/Canada: study from Korea)
Grimsrud 2020	No findings of interest. It is about the introduction of a tax on meat.
Davey 2002	No findnings of interest
Jackson 2010	No findnings of interest
Jeżewska-Zychowicz 2007	No findnings of interest
Jolley 2015	No findnings of interest
Toma 2011	No findnings of interest
Graca 2015	Not disaggregated findings
Gonzalo 2018	Not disaggregated findings (animal welfare and enviromentals)
Pavlovski 2003	Not interpretable findings
Bakker 2010	Not retrivable
NR 2011	Not retrivable
Glitsch 2001	Not retrivable

Glitsch 2000	Not retrivable
Kayser 2011	Not retrivable
Lima 2005	Not retrivable
MacKenzie 2016	Not retrivable
Nesse 2008	Not retrivable
Süllodouble 2008	Not retrivable
Boyle 2012	Opinion paper
Kutluk 2014	Power Point Presentation
Tomlinson 2015	Power Point Presentation
Carlucci 2015	Review
Corrin 2017	Review
Hamilton 2006	Review
Hartmann 2017	Review
Ingenbleek 2011	Review
Izmirli2012	Review
Joyce 2012	Review
Lyles 2014	Review
Napolitano 2010b	Review
Ruby 2012	Review
Sumpter 2015	Review
Timm 2016	Review
Walley 2014	Review
Rothgerber 2019	Study design
Begue 2019	Study design (it is an intervention/experimental study)
Carfora 2019	Study design (it is an intervention/experimental study)
Bruenner 2018	Study design: experimental/intervention study
Camilleri 2019	Study design: experimental/intervention study
Jay 2019	Study design: experimental/intervention study
Vanio 2019	Sudy design: experimental/intervention study

Reference

1. Aker C. 2004 OPC carcass evaluation program provides insight into consumer preferences. In: Centralia Swine Research Update January 26th. 2005; II-8.
2. Antunes G, Inacio AC, Martins C, Neves I, Preto A, Laires PA. Red and processed meat intake perception in the Portuguese population: Association with the who alert of 2015-preliminary results of the malert study. Elsevier Ltd. 2016; 19:A763.
3. Gardini I, Lanati EP, Caporaso N, Fontana R, Lidonnici D. Nutritional aspects and disinformation in liver disease: A questionnaire-based survey in Italian patients. Elsevier Ltd. 2015; 18: A630-A631.
4. Jensen ML, Frongillo EA, Leroy J, Blake CE. Understanding how participating in a food-aid program in rural Guatemala alters household dietary choices. 2012.
5. Lawrence A, Jones HJ. Exploring Australian's attitudes towards five food group foods and discretionary choices. Abstracts / Journal of Nutrition & Intermediary Metabolism. 2017; 8:60-121. FSTA:2017-12-Aj13081
6. Randel PF, Saadoun A, Araujo-Febres O. 60th International Congress of Meat Science and Technology, Maldonado, Uruguay, 17-22 August 2014. Asociación Latinoamericana de Producción Animal. 2014; 22:1-658.
7. Resano WVH, de Barcellos MD, Grunert KG, Pérez-Cueto FJA. Obesity and overall satisfaction with pork meat and derived pork-based products. Nutrición Hospitalaria. 2010; 25:123-126.
8. Richmond JP, Kelly MG. Examining the knowledge and perception of the lifestyle risk factors for cancer development among cancer survivors in Ireland and development of an educational tool. Elsevier Ltd. 2015; 51:165.
9. Sanderson L, Lodge JK. Exploring the characteristics of processed meat consumers and facilitators of reducing processed meat consumption. Proceedings of the Nutrition Society. 2015; 74:E316. FSTA:2017-02-Sa0547
10. Thaxton YV, Christensen KD, Mench JA, Rumley ER, Daugherty C, Feinberg B, Parker M, et al. Symposium: Animal welfare challenges for today and tomorrow. Poultry Science Association Inc. 2016; 95:2198-207. Doi: 10.3382/ps/pew099. Epub 2016 Mar 18.
11. Touillaud M, Kempf-Lepine AS, Bajard A, Gargi T, Carretier J, Foucaut AM, Berthouze-Aranda S, et al. Opinion of breast cancer patients candidates for chemotherapy on the nutrition cancer relationships. Churchill Livingstone. 2012; 7:84.
12. ICoMST. International Congress of Meat Science and Technology--ICoMST 2009. Meat Science. 2010; 84:227. doi: 10.1016/j.meatsci.2009.11.005.
13. Joo ST, Swatland HJ, Lee SK, Warner R. 56th International Congress of Meat Science and Technology, Jeju, Korea, 15-20 August 2010. Meat Science. 2010; 86:248.
14. Kowalkowska J, Babicz-Zielinska E, Wadolowska L, Czarnocinska J, Jezewska-Zychowicz M. The effect of attitudes towards health and food on female dietary patterns. The Gebahealth study.Nutrition & Metabolism. 2015; 67:107-108.
15. Gorecka D, Czarnocinska J, Idzikowski M, Kowalec J. Dietary Attitudes Of Adults In Relation To Functional Food Depending On The Age And Sex. Nauka. Technologia. Jakość. 2009; 16:320-326. WOS:000269763300040
16. Mauracher TTC, Vecchiato D. Consumer preferences regarding the introduction of new organic products. The case of the Mediterranean sea bass (Dicentrarchus labrax) in Italy. 2013; 63:84-91.

17. Napolitano F, Braghieri A, Piasentier E, Favotto S, Naspetti S, Zanoli R. Effect of information about organic production on beef liking and consumer willingness to pay. Food Quality and Preference. 2010; 21:207-212. WOS:000272752300006
18. Allen MW, Ng SH. Human values, utilitarian benefits and identification: The case of meat. European Journal of Social Psychology. 2003; 33:37-56.
19. Flight I, Russell CG, Blossfeld I, Cox DN. From sensory attributes to marketing hooks: using laddering to understand consumer perceptions of red meat. Food Australia. 2003; 55:418-424. WOS:000185053100028
20. Pourová M, Stehlík V. Czech consumers' evaluation of chosen meat products. AGRIC. ECON. 2002; 48:121-125.
21. Hartmann C, Alice G, Jing S, Michael S. Psychology of eating insects: A cross-cultural comparison between Germany and China. Food Quality Preference. 2015; 44:148-156
22. Zlaoui MO, Dhraief MZ, Rouegue TB. Determinants of broilers consumption in Tunisia: consumers' expectations and perceptions. Institut National de la Recherche Agronomique de Tunisie (INRAT). 2014; 87:91-105.
23. Nantes L, Kiefer C, Moura MS, Saraiva A, Brumatti RC, Silva CM, Silva EA. Profile and preference of pork consumers in Campo Grande. MS. Universidade Federal da Grande Dourados (UFGD). 2010; 7:460-467.
24. Sepúlveda WS, Maza MT, Pardos L. Aspects of quality related to the consumption and production of lamb meat. Consumers versus producers. Meat Science. 2011; 87:366-72. doi: 10.1016/j.meatsci.2010.11.013. Epub 2010 Nov 20.
25. Kang HK, Park KT, Jeon JJ et al. A Survey of Korean Consumers' Awareness on Animal Welfare of Laying Hens [2018]Hong, E.C., National Institute of Animal Science, Rural Development Administration, Pyeongchang, Republic of Korea.
26. Xu L, Yang X, Wu L, Chen X, Chen L, Tsai FS. Consumers' Willingness to Pay for Food with Information on Animal Welfare, Lean Meat Essence Detection, and Traceability. Int J Environ Res Public Health. 2019;16(19).
27. Yin J, Yang D, Zhang X, Zhang Y, Cai T, Hao Y, et al. Diet shift: Considering environment, health and food culture. Sci Total Environ. 2020;719:137484.
28. Lai J, Wang HH, Ortega D, Widmar N. Factoring Chinese consumers' risk perceptions into their willingness to pay for pork safety, environmental stewardship, and animal welfare. Food Control. 2018; 85: 423-431
29. Sonoda, Yuta; Oishi, Kazato; Chomei, Yosuke; Hirooka, Hiroyuki (2018). How do human values influence the beef preferences of consumer segments regarding animal welfare and environmentally friendly production?. Meat Science, (), S0309174017315036–.
30. Altintzoglou T, Verbeke W, Luten J. Association of health involvement and attitudes towards eating fish on farmed and wild fish consumption in Belgium, Norway and Spain. Aquacult Int 2011; 19:475-488.
31. V Arenas de Moreno L, Jerez-Timaure N, Valerio Hernandez J, Huerta-Leidenz N, Rodas-Gonzalez A. Attitudinal Determinants of Beef Consumption in Venezuela: A Retrospective Survey. Foods. 2020;9(2).
32. Munni US, Hossain S, Ahmed KR. Nutritional beliefs and practices among diabetic pregnant mothers in a tertiary care hospital in Bangladesh. Elsevier Ltd. 2017; 11:287-290.

33. Davaasuren L, Khamaganova IV. Analysis of consumer preference to chopped semi-finished goods from sheep meat of Mongolian breed. Food Processing: Techniques and Technology. 2017; 46(3):141-146. FSTA:2018-04-Sl1557
34. Jen MY, Wang SM. Understanding the purchasing behaviour of Taiwanese meat consumers in light of rising sustainability concerns. British Food Journal. 2015; 117:1474-1487. WOS:000354645400004
35. Delanoue E, Roguet C, Selmi A. Society protest against pig breeding in France: point of views of pig industry professionals and NGO's [Conference poster]. Institut du Porc. 2014; 46:235-240.
36. Sapp SG, Knipe CL. Japanese consumer preferences for processed pork. Agribusiness. 1990; 6:387-400.
37. Pribis P, Pencak RC, Grajales T. Beliefs and attitudes toward vegetarian lifestyle across generations. Nutrients. 2010; 2(5):523-31. doi: 10.3390/nu2050523. Epub 2010 May 17. PubMed PMID: 22254039; PubMed Central PMCID:PMC3257659.
38. Roos G, Prattala R, Koski K. Men, masculinity and food: interviews with Finnish carpenters and engineers. Appetite. 2001; 37:47-56. FSTA:2001-12-Ag2140
39. Bovell-Benjamin A, Elmubarak E. Diet-nutrition-related cancer prevention knowledge and beliefs of Sudanese in hartoum: A descriptive study. Open Journal of Preventive Medicine. 2013; 3:320-328.
40. Maskarinec G, Murphy S, Shumay DM, Kakai H. Dietary changes among cancer survivors. European Journal of Cancer Care. 2001; 10:12-20. WOS:000169353000015
41. Bonne K, Verbeke W. Muslim consumer's motivations towards meat consumption in Belgium: qualitative exploratory insights from means-end chain analysis. Anthropology of Food. Food and Religion. 2006; 11759.
42. Elmubarak E, Bromfield E, Bovell-Benjamin AC. Focused interviews with Sudanese Americans: perceptions about diet, nutrition, and cancer. Preventive Medicine. 2005; 40:502-509. WOS:000228196300002
43. Hu J, Duval Y. Qualitative analysis and implications of Chinese expatriates' food consumption behavior.Journal of Food products Marketing. 2003; 9:31-47.
44. Bohm I, Lindblom C, Abacka G, Bengs C, Hornell A. "He just has to like ham" The centrality of meat in home and consumer studies. Appetite. 2015; 95:101-112. FSTA:2016-01-Sa0166
45. Hall TE, Amberg SM. Factors influencing consumption of farmed seafood products in the Pacific northwest. Appetite. 2013; 66:1-9. DOI: 10.1016/j.appet.2013.02.012. Epub 2013 Feb 18.
46. Jaffry S, Pickering H, Ghulam Y, Whitmarsh D, Wattage P. Consumer choices for quality and sustainability labelled seafood products in the UK. Food Policy. 2004; 29:215-228.
47. Lee IO, Jung SY, Hong GJ. An analysis on nutrition knowledge level and food preference according to the food-related lifestyle tendency - focus on housewives in the Seoul and Gyeonggi area. Korean Soc. Food Cult. 2016; 31:33-41. FSTA:2016-09-Aj8126
48. Morales LE, Higuchi A. Is fish worth more than meat? - How consumers' beliefs about health and nutrition affect their willingness to pay more for fish than meat. Food Quality and Preference. 2018; 65:101-109. FSTA:2018-05-Rc1347
49. Rani ZT, Hugo A, Muchenje V. Perceptions of rural consumers on the quality of mutton in the Eastern Cape province, South Africa. Academic Journals. Academic Journals. 2013; 8:921-931.

50. Schlup Y, Brunner T. Prospects for insects as food in Switzerland: a tobit regression.Food Quality and Preference. 2018; 64:37-46. FSTA:2018-03-Sp1453
51. Bodnar K, Benak A, Skobrak EB. Analyses of consumer preferences and attitudes on Hungarian game meat market (preliminary report). Universitatea de Stiinte Agricole Si Medicina Veterinara "Ion Ionescu de la Brad".2010; 53:9-12.
52. Firetti R, Carrer CC, Silva VL, Trinidade MA, Souza SC, Savastano Junior H; Ribeiro MMLO. The perception of Sao Paulo state consumers in relation to ship meat: analysis by principal components. Rev. Bras. Saúde Prod. 2010; 11:1-13. FSTA:2011-11-Sl3459
53. Honkanen P, Olsen SO. Environmental and animal welfare issues in food choice The case of farmed fish. British Food Journal. 2009; 111:293-309. WOS:000265672700015
54. Hulya S, Aliye HM. Affecting the choice factors of fishery products consumption in Turkey. Journal of Animal and veterinary Advances 2011; 10(1):87-91.
55. Joyce SDAW, Comfort J, Hallett J. The cow in the room: public knowledge of the links between dietary choices and health and environmental impacts. Environmental. Health Insights. 2008; 1:914.
56. Pieniak Z, Verbeke W, Olsen SO, Hansen KB, Brunsa K.Health-related attitudes as a basis for segmenting European fish consumers. Food Policy. 2010; 35:448–455.
57. Pieniak Z, Verbeke W, Scholderer J. Health-related beliefs and consumer knowledge as determinants of fish consumption. Journal of human nutrition and dietetics. 2010; 23:480–488.
58. Protino J, Jacquinot M, Bouvarel I, Berri C, Magdelaine P. The place of sustainable development in consumer expectations. Institut Technique de l'Aviculture. 2013; 40.
59. Thong NT, Solgaard HS. Consumers food motives and seafood consumption. Food Quality and Preference.2016; 56:181-188
60. van Dijk H, Fischer ARH, Honkanen P, Frewer LJ. Perceptions of health risks and benefits associated with fish consumption among Russian consumers. Appetite. 2011; 56:227-34. doi: 10.1016/j.appet.2010.12.008.
61. Verbeke W, Vackier I. Individual determinants of fish consumption: application of the theory of planned behaviour. Appetite. 2005; 44:67-82
62. Verbeke W, Vanhonacker F, Sioen I, Van Camp J, De Henauw S. Perceived importance of sustainability and ethics related to fish: a consumer behavior perspective. BioOne. 2007; 36:580-5.
63. Verbeke W. Profiling consumers who are ready to adopt insects as a meat substitute in a Western society. Food Quality and Preference. 2014; 39:147-155. WOS:000342531300017
64. Stefani G, Scarpa R, Cavicchi A. Exploring consumer's preferences for farmed sea bream. Aquacult Int. 2012; 20:673-691.
65. Adamski M, Kuzacuteniacka J, Milczewska N. Preferences of consumers for choosing poultry meat. POLISH JOURNAL OF NATURAL SCIENCES. 2017; 32(2):261-271.
66. Morales LE, Higuchi A. Is fish worth more than meat? - How consumers' beliefs about health and nutrition affect their willingness to pay more for fish than meat. Food Quality and Preference. 2018; 65:101-109. FSTA:2018-05-Rc1347
67. Clonan A, Holdsworth M, Swift JA, Leibovici D, Wilson P. The dilemma of healthy eating and environmental sustainability: the case of fish. Public Health Nutrition. 2011; 15:277-284.

68. Hall TE, Amberg SM. Factors influencing consumption of farmed seafood products in the Pacific northwest. Appetite. 2013; 66:1-9. DOI: 10.1016/j.appet.2013.02.012. Epub 2013 Feb 18.
69. Jaffry S, Pickering H, Ghulam Y, Whitmarsh D, Wattage P. Consumer choices for quality and sustainability labelled seafood products in the UK. Food Policy. 2004; 29:215-228.
70. Stefani G, Scarpa R, Cavicchi A. Exploring consumer's preferences for farmed sea bream. Aquacult Int. 2012; 20:673-691.
71. Schlup Y, Brunner T. Prospects for insects as food in Switzerland: a tobit regression.Food Quality and Preference. 2018; 64:37-46. FSTA:2018-03-Sp1453
72. Emberger-Klein, A., & Menrad, K. (2018). The effect of information provision on supermarket consumers' use of and preferences for carbon labels in Germany. Journal of Cleaner Production, 172, 253–263.
73. Santos TMB, Cappi N, Simões ARP, Santos VAC, Paiano D, Garcia ERM. Diagnostic of the swine meat consumer profile in Aquidauana-MS. Escola de Medicina Veterinária, Universidade Federal da Bahia. 2011; 12:1-13.
74. Bryant, C., K. Szejda, N. Parekh, V. Desphande and B. Tse (2019). "A Survey of Consumer Perceptions of Plant-Based and Clean Meat in the USA, India, and China." Frontiers in Sustainable Food Systems 3.
75. Biermann, G. and H. Rau (2020). "The meaning of meat: (Un)sustainable eating practices at home and out of home." Appetite 153: 104730-104730.
76. Karakaya E, Incİ H. The preferences of poultry meat consumption of household in central district of Bingöl. Uludaghacek˜ Üniversitesi. 2014; 28:53-64.
77. McCarthy M, de Boer M, Henson J. Irish consumer perceptions of meat hazards and use of extrinsic information cues. Taylor & Francis Ltd. 2004; 1:99-107.
78. Clonan A, Holdsworth M, Swift JA, Leibovici D, Wilson P. The dilemma of healthy eating and environmental sustainability: the case of fish. Public Health Nutrition. 2011; 15:277-284.
79. Ali A, Kassim B, Hasan SAJ, Manap YA, Sazili AQ. Effects of feeding the herb Borreria latifolia on the meat quality of village chickens in Malaysia. Agricultural Science Digest. 2017; 96:1767-1782. FSTA:2018-05-Sn2163
80. Allen MW, Wilson M, Ng SH, Dunne M. Values and beliefs of vegetarians and omnivores. The Journal of Social Psychology. 2000; 140:405-422. WOS:000088944500001
81. Altintzoglou T, Einarsdottir G, Valsdottir T, Schelvis R, SkÃra T, Luten J. A voice-of-consumer approach in development of new seafood product concepts. Journal of Aquatic Food Product Technology. 2010a; 19:130-145.
82. Altintzoglou T, Hansen KB, Valsdottir T, Odland JO, Martinsdottir E, Brunso K, Luten J. Translating barriers into potential improvements: The case of new healthy seafood product development. Journal of Consumer Marketing. 2010b; 27:224-235.
83. Andrade JC, Deliza R, Yamada EA, Galvão MTEL, Frewer LJ, Beraque NJ. Consumer perception of risks associated with food, safety and traceability. Campinas. 2013; 16:184-191.
84. Apostolidis C, McLeay F. Should we stop meating like this? Reducing meat consumption through substitution. Food Policy. 2016; 65: 74-89. FSTA:2017-03-Sa1092
85. Apple RD. Cancer from beef: DES, federal food regulation, and consumer confidence by Alan Marcus, Review by Rima D. Apple. Technology and Culture. 37:396-397. WOS:A1996VP42700036

86. Aral Y, Aydin E, Demir P, Akin AC, Cevger Y, Kuyululu CYK, Arikan MS. Consumer preferences and consumption situation of chicken meat in Ankara Province, Turkey. Turkish Journal of Veterinary and Animal Sciences. 2013; 37:582-587. WOS:000323607100017
87. Arica SÇ. Determination of consumption habits and preferences of fishery products in Hatay Province, the people of İskenderun. Su Ürünleri Merkez Araștırma Enstitüsü. 2017; 17:233-243.
88. Armağan G, Özdogan M. Ekolojik Yumurta ve Tavuk Etinin Tüketim Eğilimleri ve Tüketici Özelliklerinin Belirlenmesi. Hayvansal Üretim. 2005; 46(2):14-21.
89. Arthur R, Nichols S. Nutritional knowledge, attitudes and dietary behaviour among state employees in trinidad and tobago. Oxford University Press. 2011; 173:32.
90. Azevedo BAR, Roediger MdA, Szarfarc SC. Perception of the causes and risks of anaemia on pregnant women attending the Family Health Strategy Program in São Caetano do Sul (São Paulo).Nucleo de Estudos e Pesquisas em Alimentacao. Segurança Alimentar e Nutricional, Campinas. 2016; 23(2):984-992.
91. Badertscher-Fawaz R. Demand for meat from particularly animal friendly production systems. 1998; 5:57-60.
92. Balcombe K, Bradley D, Fraser I, Hussein M. Consumer preferences regarding country of origin for multiple meat products. Food Policy. 2016; 64:49-62. WOS:000388060900005
93. Ballantyne W. A Proactive Approach to Animal Welfare. Advances in Pork Production. 2006; 17:139-144.
94. Baron PJ, Carpenter EM. A review of consumer attitudes and requirements for meat. Department Of Agricultural Marketing University Of Newcastle Upontyne. 1976; 23.
95. Bazell M. Strife among the ''Table-Fellows'' + Meat consumption and abstinence as expressed in the historical religious context of the Middle-Ages: Conflicting attitudes of early and medieval Christians toward the eating of meat. Journal of the American Academy of Religion. 1997; 65:73-99. WOS:A1997WX57100004
96. Bennett R, Blaney R. Social consensus, moral intensity and willingness to pay to address a farm animal welfare issue. Journal of Economic Psychology. 2002a; 23:501-520.
97. Bennett RM, Marchello JA, Gorman WD, Ribal JC. Consumer acceptance of beef from steers fed low, medium or high concentrate diets.2002b; 49: FSTA:1980-07-S-1220
98. Bennett RM. Farm animal welfare and food policy. Food Policy. 1996; 22:281-288.
99. Bergstra T, Hogeveen H, Kuiper WE, Lansink A, Stassen EN. Attitudes of Dutch Citizens toward Sow Husbandry with Regard to Animals, Humans, and the Environment. Anthrozoös. 2017; 30:195-211. WOS:000401518300002
100. Berndsen M, Pligt van der J. Ambivalence towards meat. Appetite. 2004; 42:71-78.
101. Berndsen M, Pligt van der J. Risks of meat: the relative impact of cognitive, affective and moral concerns.Appetite. 2005; 44:195–205.
102. Betts NM, Amos RJ, Georgiou C, Hoerr SL, Ivaturi R, Keim KS, Tinsley A, et al. What young adults say about factors affecting their food intake. Ecology of Food and Nutrition. 1995; 34:
103. Blasco M, Maza MT, Campo MM. Consumers's attitudes towards lamb meat with quality mark. Asociación Interprofesional para el Desarrollo Agrario. AIDA. 2017; 17:726-728.
104. Blom H, Nerbrink E, Dainty R, Hagtvedt T, Borch E, Nissen H, Nesbakken T Accelerated Production of Dry Fermented Sausage. Meat Science. 1996; 43:229-242

105. Bovell-Benjamin AC, Dawkin N, Pace RD, Shikany JM. Use of focus groups to understand African-Americans' dietary practices: Implications for modifying a food frequency questionnaire. Prev Med. 2009; 48:549-554.
106. Bratanova B, Loughnan S, Bastian B. The effect of categorization as food on the perceived moral standing of animals. Appetite. 2011; 57:193-196.
107. Breukink HR, Casey NH. ASSESSING THE ACCEPTABILITY OF PROCESSED GOAT MEAT. S.-Afr.Tydskr.veek. 1989; 19(2):76-80. WOS:A1989AH28400005
108. Burger J, Gochfeld M. Perceptions of the risks and benefits of fish consumption: Individual choices to reduce risk and increase health benefits. Environ Res. 2009; 109:343-349.
109. Butterworth W. Science and society improving animal welfare. Welfare Quality conference proceedings; 2005 Nov 17-18; Brussels, Belgium. 2005.
110. Büyükkaragöz, Bas M, Saglam D, Cengiz SE. Consumers' awareness, acceptance and attitudes towards functional foods in Turkey. International Journal of Consumer Studies. 2014; 38:628-635. WOS:000344323900008
111. Caklová K, Ulčák Z. Organic meat consumers and their awareness of animal welfare as reflected by purchase preferences. University of East Sarajevo, Faculty of Agriculture. 2014; 958-964.
112. Campbell J, Macdiarmid JI, Douglas F. Young people's perception of the environmental impact of food and their willingness to eat less meat for the sake of the environment: a qualitative study. Proceedings of the Nutrition Society. 2016; 75: FSTA:2017-03-Sa1339
113. Capie F. Consumer Preference: Meat in England and Wales.Consumer Preference. 1976; 85-94
114. Carlsson F, Frykblom P, Lagerkvist CJ. Consumer benefits of labels and bans on GM foods - choice experiments with Swedish consumers. European Review of Agricultural Economics. 34(3):321-344.
115. Cerjak M, Karolyi D, Mesić Z. Consumers' attitudes towards farm animal welfare and their influence on meat consumption. Agronomski Fakultet, Sveučilišta u Zagrebu. 2011; 76:283-286.
116. Cheah, Isaac; Sadat Shimul, Anwar; Liang, Johan; Phau, Ian (2020). Drivers and barriers toward reducing meat consumption. Appetite, (), 104636–.
117. Christensen T, Mørkbak M, Hasler B, Lundhede T, Porsbo LJ, Christoffersen LB. Information, risk perception and consumer behaviour - a choice experiment on food safety and animal welfare. Fødevareøkonomisk Institut. 2006; 267.
118. Clonan A, Wilson P, Swift JA, Leibovici DG, Holdsworth M. Red and processed meat consumption and purchasing behaviours and attitudes: impacts for human health, animal welfare and environmental sustainability. Public Health Nutrition, 2015; 18(13):2446–2456.
119. Corcoran K, Bernués A, Manrique E, Pacchioli T, Baines R, Boutonnet JP. Current consumer attitudes towards lamb and beef in Europe. Centre International de Hautes Etudes Agronomiques Méditerranéennes. CIHEAM. 2001; 75-79.
120. Cox DN, Anderson AS, Reynolds J, McKellar S, Lean MEJ, Mela DJ. Take five, a nutrition education intervention to increase fruit and vegetable intakes: impact on consumer choice and nutrient intakes. British Journal of Nutrition. 1998; 80:123-131

121. Dacosta KO, Wilson JF. Food preferences and eating attitudes in three generations of black and white women. Appetite. 1996; 27:183-191. WOS:A1996VT21700007
122. Dalen GA. Assuring Eating Quality of Meat. Meat Science. 1996; 43:21-33.
123. De Boer ADWJ, Aiking H. Help the climate, change your diet: A cross-sectional study on how to involve consumers in a transition to a low-carbon society. Appetite. 2016; 98:19-27.
124. De Houwer J, De Bruycker E. Implicit attitudes towards meat and vegetables in vegetarians and nonvegetarians. International Journal of Psychology. 2007; 42:158-165. WOS:000247177300003
125. De Jonge J, van Trijp H. Heterogeneity in consumer perceptions of the animal friendliness of broiler production systems. Food Policy. 2014; 49:174-185. WOS:000347760400018
126. De Jonge J, van Trijp HCM. The impact of broiler production system practices on consumer perceptions of animal welfare. Poultry Science. 2013; 92:3080-3095. WOS:000330526500002
127. De Morais Sato P, da Rocha Pereira P, de Carvalho Stelmo I, Unsain RF, Ulian MD, Sabatini F, Martins PA, et al. Eating practices and habitus in mothers. A Brazilian population-based survey. Appetite. 2014;82:16-28.
128. Deimel I, Böhm J, Schulze B. Low meat consumption as a precursor to vegetarianism? Qualitative study on motivational structures for low meat consumption. Institut für Agrarökonomie der Universität Göttingen. 2010; 41 pp. ISSN 1865-2697
129. Del Coz JJ, Diez J, Bahamonde A, Sanudo C, Alfonso M, Berge P, Dransfield E, et al. Learning the reasons why groups of consumers prefer some food products.2006; 4065:297-309. WOS:000239623700024
130. Dentoni D, Tonsor G, Calantone R, Peterson HC. "Animal Welfare" Practices along the Food Chain: How Does Negative and Positive Information Affect Consumers? 113th EAAE Seminar "A resilient European food industry and food chain in a challenging world". Chania, Crete, Greece, 03-06 September 2009.
131. Derell H, Çelİk R, Saygı H, Tekİnay AA. A study on the sea food consumption and consumer preferences in Manisa Province. Su Ürünleri Merkez Araștırma Enstitüsü.2016; 16:115-128.
132. Dockès AC, Magdelaine P, Daridan D, Guillaumin A, Rémondet M, Selmi A, Gilbert H, et al. Attentes En Matière D'élevage Des Acteurs De La Sélection Animale, Des Filières De L'agroalimentaire Et Des Associations. INRA Prod. Anim. 2011; 24:285-296.
133. Dreezens E, Martijn C, Tenbult P, Kok G, Vries NK. Food and the relation between values and attitude characteristics. Appetite. 2005; v. 45:
134. Ebewore SO, Ovharhe OJ, Emaziye PO. Acceptability of bush meat as a source of animal protein in Delta State, Nigeria: implication for extension services. Northeast Agricultural University. 2015; 22:67-78.
135. EFSA (European Food Safety Authority). EFSA opinion on the welfare of beef cattle. doi: 10.1136/vr.e3635: 2012.
136. EFSA (European Food Safety Authority). Scientific Opinion on the substantiation of health claims related to meat or fish and the improvement of non-haem iron absorption (ID 1223) pursuant to Article 13(1) of Regulation (EC) No 1924/2006. 9: 2011.

137. El-Abbadi N, Peters C, Griffin T, Nelson M, Jacques P. Development of a dietary environmental index to assess nutritional quality versus environmental effect of foods and dietary patterns. Meeting abstract. 2017; FSTA:2017-11-Aj11457
138. El-Naga SA. An analytical study for the development of consumers' expenditure and consumption of animal products in Egypt (challenges-opportunities). Selcuk University.2015; 206-212.
139. EuropeanCommission (2007). Attitudes of EU citizens towards Animal Welfare: Fieldwork September-October 2006. Special Eurobarometer. 2007; Wave 66.1
140. EuropeanCommission. Attitudes of consumers towards the welfare of farmed animals: Fieldwork : February- March 2005. Special Eurobarometer. 2005; Wave 63.2.
141. Feeley KJ, Machovina B. Increasing preference for beef magnifies human impact on world's food web.PNAS. 2014; 111:E794. doi: 10.1073/pnas.1323071111. Epub 2014 Feb 4.
142. fish consumption: A cross-cultural study. Appetite. 2008; 49:84-91.
143. Fishman LE. Just feed me the sausage: one congressional staffer's view. The Milbank Quarterly. 2003; 81:143-6.
144. Fotea L, Costăchescu E, Leonte D, Nistor CE. Research regarding consumer preferences in acquiring fish and fish products. University of Agricultural Sciences and Veterinary Medicine Iasii. 2012; 58:112-115.
145. Frewer LJ, Kole A, Van De Kroon SMA, De Lauwere C. Consumer attitudes towards the development of animal-friendly husbandry systems. Journal of Agricultural and Environmental Ethics. 2005; 18:345-367. WOS:000231759600003
146. Gill CO. Extending the Storage Life of Raw Chilled Meats. Meat Science, 1996; 33:884-892. WOS:A1996VQ85000023
147. Gittelsohn AEVJ. Sociocultural and household factors impacting on the selection, allocation and consumption of animal source foods: current knowledge and application. American Society for Nutritional Sciences. 2003; 133:4036S-4041S.
148. Graca J, Calheiros MM, Oliveira A. Attached to meat? (Un)Willingness and intentions to adopt a more plant-based diet. Appetite. 2015; 95:113-125. FSTA:2016-01-Sa0167
149. Gregory NG. Welfare and Hygiene during Preslaughter Handling. Meat Science. 1996; 43:35-46.
150. Grunert KG, Bredahl L, Brunsø K. Consumer perception of meat quality and implications for product development in the meat sector. Meat Science.2004; 66:259-272.
151. Gutkowska K, Żakowska-Biemans S, Sajdakowska M, Kosicka-Gębska M, Wierzbicki J. Perception of beef attributes by Polish consumers as a factor determining demand for this food category. Oeconomia. 2011; 10(4):33-41.
152. Hamlin R. Functional or constructive attitudes: Which type drives consumers' evaluation of meat products?. Meat Science. 2016; 117:97-107
153. Hargin KD. Authenticity issues in meat and meat products. Meat Science. 1996; 43S1:277-89.
154. Hayley A, Zinkiewicz L, Hardiman K. Values, attitudes, and frequency of meat consumption. Predicting meat-reduced diet in Australians. Appetite. 2015; 84:98-106. FSTA:2015-03-Sa0747
155. Heid A, Hamm U. Consumer acceptance of alternatives to piglet castration without anaesthesia in organic farming.In: Organic is Life Knowledge for Tomorrow: Volume 2 Socio-Economy, Livestock, Food Quality, Agro-Ecology and Knowledge Dissemination. Proceedings

of the Third Scientific Conference of the International Society of Organic Agriculture Research (ISOFAR). ISOFAR. 2011. 14-17.
156. Heleski MCR, Zanella AJ. Assessing attitudes toward farm animal welfare: a national survey of animal science faculty members. American Society of Animal Science. 2004; 82:2806-14.
157. Heleski R, Mertig AG, Zanella AJ. Stakeholder attitudes toward farm animal welfare. Anthrozoös. 2006; 19:290-307. WOS:000244696500001
158. Henchion M, Hayes M, Mullen AM, Fenelon M, Tiwari B. Future protein supply and demand: strategies and factors influencing a sustainable equilibrium. Foods. 2017; 6:53.
159. Herzog HA, Golden LL. Moral Emotions and Social Activism: The Case of Animal Rights. Journal of Social Issues. 2009; 65:485-498.
160. Hilverda F, Jurgens M, Kuttschreuter M. Word associations with "organic": what do consumers think of?. British Food Journal. 2016; 118:2931-2948. WOS:000390692100006
161. Hobbs SH. Attitudes, practices, and beliefs of individuals consuming a raw foods diet. Explore. 2005; 53:47-65. WOS:000227638600003
162. Holt V, Nordstrom J, Kohrs MB. Food preferences of older adults. Journal of Nutrition for the Elderly. 1987; 6(3):47-56,
163. Hong H, Lee J. Survey on Korean food preference of college students in Seoul - focused on the staple food and snack. Korean Nutrition Society. 2006; 39:699-706.
164. Hovi M, Martini A, Padel S. Socio-economic aspects of animal health and food safety in organic farming systems. Proceedings of the 1st SAFO Workshop, Florence, Italy, 5-7 September 2003. University of Reading. 2003.
165. Hughes D. Animal welfare: the consumer and the food industry. British Food Journal. 1995; 97:3-7. FSTA:1996-04-D-0014
166. Hung Y, de Kok TM, Verbeke W. Consumer attitude and purchase intention towards processed meat products with natural compounds and a reduced level of nitrite. Meat Science. 2016 121:119-126.
167. Hunter E, Röös E. Fear of climate change consequences and predictors of intentions to alter meat consumption. Food Policy. 2016; 62:151-160. WOS:000380417200014
168. Hygreeva D, Pandey MC, Radhakrishna K. Potential applications of plant based derivatives as fat replacers, antioxidants and antimicrobials in fresh and processed meat products. Meat Science. 2014; 98:47-57. FSTA:2014-09-Sa6564
169. Inoue M, Iwasaki M, Otani T, Sasazuki S, Tsugane S. Public awareness of risk factors for cancer among the Japanese general population: A population-based survey. BMC Public Health. 2006; 6(2). WOS:000234890000001
170. Ippolitov S. "I Cut Some Camel Meat ...": Hungry Daily Life and Gastronomic Preferences of Russian Emigrants (1920-1930s). Anti-Bolshevik Russia. 2015; 82-99. WOS:000370651700006
171. Jackson P, Ward N, Russell P, Goodman MK, Goodman D, Redclift M. Manufacturing Meaning along the Chicken Supply Chain: Consumer Anxiety and the Spaces of Production. Consuming Space. 2010; WOS:000337353300009
172. Joseph RL. Very Fast Chilling of Beef and Tenderness-a Report From an EU Concerted Action. Meat Science. 1996; 43:217-227.
173. Juma GP, Ngigi M, Baltenweck I, Drucker AG. Consumer demand for sheep and goat meat in Kenya. Small Ruminant Research. 2010; 90:135-138. WOS:000277882500021

174. Kalof L, Diets T, Stern PC, Guagnano GA. Social Psychological and Structural Influences on Vegetarian Beliefs. Rural Sociology. 1999; 64:500-511.
175. Kenyon PM, Barker ME. Attitudes towards meat-eating in vegetarian and non-vegetarian teenage girls in England - an ethnographic approach. Appetite. 1998; 30:185-198. FSTA:1999-02-Sa0201
176. Kjaernes U, Miele M, Roex J. Attidudes of consumers, Retailers and Producers to Farm Animal Welfare. Welfare Quality Reports. 2007.
177. Klöckner AC. A stage model as an analysis framework for studying voluntary change in food choices - The case of beef consumption reduction in Norway. Appetite. 2016; 108:434-449.
178. Knight S, Vrij A, Cherryman J, Nunkoosing K. Attitudes towards animal use and belief in animal mind. Purdue University Press. 2004; 17:43-62.
179. Koizumi S, Kobayashi S, Pan I, Takaku S, Nishino M, Nagano M. Study on consumer behavior for meat consumption in Japan (Tokyo, Osaka).Japanese Society of Animal Science. 2000; 71:621-633.
180. Koritar P,. Alvarenga M. Attitudes toward beef among nutrition college students. S. Karger AG. 2017; 71:1271.
181. Kottke V, Damm H, Fischer A, Leutz U. Engineering Aspects in Fermentation of Meat Products. Meat Science. 1996; 43:243-255.
182. Krystallis A, de Barcellos MD, Kugler JO, Verbeke W, Grunert KG. Attitudes of European citizens towards pig production systems. Livestock Science. 2009; 126:46-56. FSTA:2010-01-Sj0007
183. Kulkarni VV, Kanagaraju P, Chandirasekaran V. Survey on the knowledge level of the consumers about meat and health. Journal of Vererinary Public Health. 2009; 7:33-40. FSTA:2010-07-Sa2157
184. Kumar N, Sanjeev K. Study of consumers' behavior for non-vegetarian products in emerging market of India. Emerald Group Publishing Ltd. 2014;4:59-77.
185. Lagerkvist CJ, Carlsson F, Viske D. Swedish Consumer Preferences for Animal Welfare and Biotech: A Choice Experiment. AgBioForum. 2006; 9:51-58.
186. Lassen AD, Lehmann C, Andersen EW, Werther MN, Thorsen AV, Trolle E, et al. Gender differences in purchase intentions and reasons for meal selection among fast food customers - opportunities for healthier and more sustainable fast food. Food Quality and Preference. 2016; 47:123-129. FSTA:2016-01-Ge0176
187. Lennernas M, Fjellstrom C, Becker W, Giachetti I, Schmitt A, Winter AMR, Kearney M. Influences on food choice perceived to be important by nationally-representative samples of adults in the European Union. European Journal of Clinical Nutrition. 1997; 51:8-15
188. Lepore JR. Factors Affecting Acceptability And Identification Of Puréed Foods. Abstract of Thesis Presented to the Graduate School of the University of Florida in Partial Fulfillment of the Requirements for the Degree of Master of Science. 2013.
189. Liljenstolpe C. Evaluating animal welfare with choice experiments: An application to Swedish pig production. Agribusiness. 2008; 24: 67-84. WOS:000252453200005
190. Longdell GR. Recent Developments in Sheep and Beef Processing. Meat Science. 1996; 43:165-174.

191. Lopez Osornio MM, Hough G, Salvador A, Chambers EI, McGraw S, Fiszman S. Beef's optimum internal cooking temperature as seen by consumers from different countries using survival analysis statistics. Food Quality and Preference. 2008; 19(1):12-20
192. Loughnan S, Haslam N, Bastian B. The role of meat consumption in the denial of moral status and mind to meat animals.Appetite. 2010; 55:156-9. Doi: 10.1016/j.appet.2010.05.043. Epub 2010 May 19.
193. Maguire K. Perceptions of meat and food: some implications for health promotion strategies. British Food Journal. 1994; 96:11-17.
194. Margetts M, Martinez JA, Saba A, Holm L, Kearney M. Definitions of 'healthy' eating: a pan-EU survey of consumer attitudes to food, nutrition and health. European Journal of Clinical Nutrition. 1997; 51:23-29
195. Marin D, Csaholczi A, Petroman C, Petroman I. Study regarding the assessment of consumer behavior of poultry meat in Timis county, Romania. Abstracts / Journal of Biotechnology. 2016; 231:51. WOS:000380240300155
196. Matwiejczyk L, Mroz SS, Jackson K, Jarrett J, Barbossa C. "Think-Aloud" method reveals how dietitians make decisions when food shopping. Nutrition & Dietetics. 2016; 73:51-97. FSTA:2016-11-Aj10139
197. Mayfield LE, Bennett RM, Tranter RB, Wooldridge MJ. Consumption Of Welfare-Friendly Food Products In Great Britain, Italy And Sweden, And How It May Be Influenced By Consumer Attitudes To, And Behaviour Towards, Animal Welfare Attributes. International Journal of Sociology of Food and Agriculture. 2007; 15(3):59-73.
198. McIntosh WA, Fletcher RD. Factors Associated with Sources of Influence/Information in Reducing Red Meat by Elderly Subjects. Appetite. 1995; 31:285-295. WOS:A1994PG83500005
199. McIntyre, Baid A. Indulgent snack experience attributes and healthy choice alternatives. Journal of Toxicology and Environmental Health, Part A. 2009; v. 111:
200. McKendree MGS, Croney CC, Widmar NJO. BIOETHICS SYMPOSIUM II: current factors influencing perceptions of animals and their welfare. American Society of Animal Science. 2014; 92:1821-1831.
201. Menzie EL, Gordin Z, Archer TF. Consumer attitudes, knowledge and buying habits relative to beef: an Arizona case study. College of Agriculture, University of Arizona (Tucson, AZ). 1976.
202. Mijatović D, Mikuš T, Mesić Z, Njari B, Fleck ZC, Kozačinski L. Consumer opinion on influence of animal welfare to meat quality during processing. Zadružna Štampa. MESO. 2012; 14:328-332, 355.
203. Moen DS, Capps O. A nonparametric analysis of consumer preferences for fresh meat products. Journal of Food Distribution Research. 1989; 19:15-19.
204. Muringai V, Goddard E. Long-term impacts of bovine spongiform encephalopathy on beef risk perceptions and risk attitudes in Canada. Journal of Toxicology and Environmental Health, Part A2016; 79:746–761.
205. Nanni Costa L, Zambonelli P, Russo V. Proceedings of the 6th International Symposium on the Mediterranean Pig, Messina, Capo d'Orlando, Italy, 11-13 October, 2007. Facoltà di Agraria, Universita di Bologna.2007.
206. Narchi I, Walrand S, Boirie Y, Rousset S. Emotions generated by food in elderly french people. The Journal of Nutrition, Health & Aging. 2008; 12:626-633.

207. Nath J. Gendered fare? A qualitative investigation of alternative food and masculinities. Journal of Sociology. 2010; 47:261-278.
208. Neo H. Ethical consumption, meaningful substitution and the challenges of vegetarianism advocacy. Wiley-Blackwell. 2016; 182:201-212.
209. Ngapo TM, Martin JF, Dransfield E. Consumer choices of pork chops: results from three panels in France. Meat Science. 2003; 15:349-359. WOS:000221033100006
210. Nielsen HB, Sonne AM, Grunert KG, Banati D, Pollák-Tóth A, Lakner Z, Olsen NV, et al. Consumer perception of the use of high-pressure processing and pulsed electric field technologies in food production. Appetite. 2009; 52:115-126
211. Ogut S, Polat M, Orhan H, Kucukoner E. Sociodemographic status and nutrition choice of elderly living in nursing home in Isparta and Burdur. Geriatrics Society. 2008; 11:82-87.
212. Olewnik-Mikolajewska A, Guzek D, Glabska D, Sajdakowska M, Gutkowska K. Fodder enrichment and sustaining animal well-being as methods of improving quality of animal-derived food products, in the aspect of consumer perception and acceptance. Animal Science Papers and Reports. 2016; 34:361-372. WOS:000386245900006
213. Oliveros ES, Perez GT, Gonzalez-Rebeles C, Maldonado FG. Consumer attitudes and perceptions towards sustainable animal production attributes in Mexico City. Vet. Mèx. 2012; 43:87-101. WOS:000304731200001
214. Olsen SO, Heide M, Dopico DC, Toften K. Exploring the relationship between convenience and
215. O'Mahony R, Cowan C, Keane M. Dublin consumers and pork: attitudes to quality. Leanness is by far the most important quality factor for consumers. British Food Journal. 1995, 97:26-33.
216. O'Rourke K. Animal welfare: status and perception. JAVMA. 2004; 224:1565-1569.
217. Oteku IT, Igene JO, Yessuf IM. An assessment of the factors influencing the consumption of duck meat in Southern Nigeria. Pakistan Journal of Nutrition. 2006; 5:474-477.
218. Ozawa T, Lopez-Villalobos N, Blair HT. A survey of goat meat acceptability in Japan. New Zealand Society of Animal Production. 2004; 64:208-211.
219. Özen R, Yaşar A, Armutak A, Bayrak S, Gezman A, Şeker I. Attitudes of Turkish Veterinary Students and Educators towards the Moral Status of Animals and Species Rating. Kafkas Üniv Vet Fak Derg. 2009; 15(1):111-118.
220. Patil SR, Morales R, Cates S, Anderson D, Kendall D. Application of meta-analysis in food safety consumer research to evaluate consumer behaviours and practices. Journal of Food Protection. 2004; 67(11):2587–2595:
221. Paul ES, Podberscek AL. Veterinary education and students' attitudes towards animal welfare. Veterinary Record. 2000; 146:269-272.
222. Petroka K, Dinu M, Hoover C, Casini C, Sofi F. Short-term exposure to a Mediterranean environment influences attitudes and dietary profile in U.S. college students: the MEDiterranean Diet in AMEricans (A-MED-AME) pilot study. Journal of the American College of Nutrition. 2016; 35:621-626. FSTA:2017-02-Aj1649
223. Pfeiler TM, Egloff B. Examining the "Veggie" personality: results from a representative German sample. Appetite. 2017; 120:246-255. FSTA:2018-03-Sa1243

224. Pfeiler TM, Egloff B. Personality and attitudinal correlates of meat consumption: Results of two representative German samples. Appetite. 2018; 121: 294-301.
225. Phillips JC, Izmirli S.The effects of level of support for animal protection organisations on attitudes to the use of animals and other social issues. Universities Federation for Animal Welfare (The Old School, Brewhouse Hill, Wheathampstead, Herts AL4 8AN, United Kingdom). 2012; 21:583-592.
226. Piqueras-Fiszman B, Jaeger SR. Consumer segmentation as a means to investigate emotional associations to meals. Appetite. 2016; 105:249-258
227. Pirsich W, Theuvsen L. Animal welfare as a differentiation strategy for butchers. Austrian Society of Agricultural Economics. 2016; 25:107-116.
228. Popescu A. Research on consumer behaviour on Bucharest meat market. Scientific Papers Series Management , Economic Engineering in Agriculture and Rural Development. 2013; 13(1):301-307.
229. Popluga D, Spogis K, Eglite A. Future of Beef Market in Latvia - Consumers Issues and Demand. 2010; 66-73. WOS:000289965100008
230. Povey R, Wellens B, Conner M. Attitudes towards following meat, vegetarian and vegan diets: an examination of the role of ambivalence. Appetite. 2001; 37:15-26. FSTA:2001-12-Ag2138
231. Preylo BD, Arikawa H. Comparison of vegetarians and non-vegetarians on pet attitude and empathy. Anthrozoös. 2008; 21:387-395.
232. Prunty J, Apple KJ. Painfully Aware: The Effects of Dissonance on Attitudes toward Factory Farming. Anthrozoös. 2013; 26:265-278. WOS:000320483000008
233. Pufpaff KM. Breaking down the barriers between halal slaughter and animal welfare. Asociación Latinoamericana de Producción Animal Welfare. 60th International Congress of Meat Science and Technology, 17-22nd August 2014, Punta del Este, Uruguay. 2014.
234. Qing P, Xi AQ, Hu WY. Consumer Preference for Meat in China: A Case Study of Beijing. Emerging Markets Finance and Trade. 2004; 50:135-143. WOS:000341381100010
235. Queiroz MLV, Filjo JADB, Albiero D, Freitas D, Melo RPM. Consumer perception about welfare of livestock in Fortaleza, Ceara, Brazil. Revista Ciência Agronômica. 2014; 45:379-386. WOS:000336966400020
236. Radder L. Understanding consumer decision-making in adopting wild venison: a suggested framework. Food Products Press. 2003; 9:15-29.
237. Radman M, Camanzi L, Kolega A. Preferences and buying behaviour of beef consumers in Tuscany. Faculty of Agriculture. Journal of Central European Agriculture. 2005; 6:99-105.
238. Raghavendra HN, Mahajanashetti SB, Karnool NN, Kulkarni VS, Basavaraj H. Meat consumption pattern and its preference in Dharwad district: a socio-economic analysis. University of Agricultural Sciences. 2009; 22:353-358.
239. Raineri C, Antonelli R, Nunes BCP, de Barros CS, Morales AMT, Gameiro AH. Contribution to economic evaluation of systems that value animal welfare at farm. Revista Colombiana de Ciencias Pecuarias. 2012; 25:123-134. WOS:000305934800014
240. Rathod P, Landge S, Nikam TR, Hatey AA. Preferential poultry meat consumption and cooking patterns in Bijapur district of Karnataka. Journal Of Veterinary Public Health. 2011; 9(1):39-42.

241. Rothgerber H. Can you have your meat and eat it too? Conscientious omnivores, vegetarians, and adherence to diet. Appetite. 2015b; 196-203. FSTA:2015-03-Ag1684
242. Rothgerber H. Horizontal hostility among non-meat eaters. PLOS ONE. 2014; 9(5): e96457-e96457. FSTA:2014-10-Aa13375
243. Rothgerber H. Underlying differences between conscientious omnivores and vegetarians in the evaluation of meat and animals. Appetite. 2015a; 87:251-258. FSTA:2015-05-Sa1632
244. Rousset S, Deiss V, Juillard E, Schlich P, Droit-Volet S. Emotions generated by meat and other food products in women. British Journal of Nutrition. 2005; 94:609-619.
245. Ruby MB, Alvarenga MS, Rozin P, Kirby TA, Richer E, Rutsztein G. Attitudes toward beef and vegetarians in Argentina, Brazil, France, and the USA. Appetite. 2016; 96:546-554.
246. Rurik I. Nutritional differences between elderly men and women. Nutrition & Metabolism. 2006; 50:45-50. FSTA:2006-04-Aj0991
247. Rutsaert P, Barnett J, Gaspar R, Marcu A, Pieniak Z, Seibt B, Lima ML, et al. Beyond information seeking: Consumers' online deliberation about the risks and benefits of red meat. Food Quality and Preference. 2015; 39:191-201. WOS:000342531300022
248. Santos AS, Palheta IC, dos Santos RP, Nascimento TD, Melo JD. Frequency and Preference of beef meat Consumption, Swine and Poultry in Practitioners of Physical Exercise on the Academies Redencao-Para. Revista Brasileira de Nutrição Esportiva. 2017; 11:87-92. WOS:000391422600012
249. Santos S, Vilela S, Padrao P, Caraher M. Sex-related dietary changes of Portuguese university students after migration to London, UK. Nutrition & Dietetics. 2015; 72:340-346. FSTA:2016-04-Aj3231
250. Schaly LM, Oliveira MC, Salviano PAP, Abreu JM. Consumer perception about animal welfare in Rio Verde, GO. Pubvet. 2010; 38(4).
251. Scott L, Downey RG. Types of food aversions: animal, vegetable, and texture. The Journal of Psychology. 2007; 141: 127-34.
252. Scozzafava G, CasiniL, Contini C. Analysis of Italian consumer preferences for beef. NEW MEDIT.2014; 13:66-72. WOS:000335276200009
253. Segovia-López E, Albornoz-Gotera A, Ortega-Soto L. Factors that determine the selection of the beef purchase place in Maracaibo, Venezuela. Facultad de Ciencias Veterinarias, Universidad del Zulia. 2007; 17:366-371.
254. Seker I, Ozen A, Guler H, Seker P, Ozden I. Red Meat Consumption Behavior in Elazig and Consumers' Opinion in Animal Welfare. Kafkas Univ Vet Fak Derg. 2011; 17:543-550. WOS:000292686100005
255. Serpell JA. Factors Influencing Veterinary Students'Career Choices and Attitudes to Animals. Journal of Veterinary Medical Education. 2005; 32:491-496
256. Shan LC, Regan Á, Monahan FJ, Li C, Lalor F, Murrin C, Wall G, et al. Consumer preferences towards healthier reformulation of a range of processed meat products. Emerald Publishing. British Food Journal. 2017; 119:2013-2026.
257. Shepherd R. Healthier eating in the UK: the Eating, Food and Health LINK programme. Trends in Food Science & Technology. 2001; 12:425-428. FSTA:2003-02-Aj0259
258. Shipman D, Durmus B.The effect of culture on food consumption; a case of special religious days in Turkey. Canadian Center of Science and Education. Journal of Food Research. 2017; 6(2):92-99.

259. Siegrist M, Visschers VHM, Hartmann C. Factors influencing changes in sustainability perception of various food behaviors: Results of a longitudinal study. Food Quality and Preference. 2015; 46:33-39. WOS:000361255900005
260. Sigman-Grant MJ. Food choice: balancing benefits and risks. Journal Of The American Dietetic Association. 2008; 108(5):778-782. Doi: 108: 778-80. doi: 10.1016/j.jada.2008.02.024.
261. Simoes JS, Mársico ET, da Cruz AG, de Freitas MQ, Doro LH, Adam C. Effect of sustainability information on consumers' liking of freshwater prawn (Macrobrachium rosenbergii). J Sci Food Agric. 2015; 95:3160-4. doi: 10.1002/jsfa.7055. Epub 2015 Feb 9.
262. Song HJ, Simon JR, Patel DU. Food Preferences of Older Adults in Senior Nutrition Programs. Journal of Nutrition in Gerontology and Geriatrics. 2014; 33:55-67
263. Sow TMA, Grongnet JF. Sensory characteristics and consumer preference for chicken meat in Guinea. Poultry Science. 2010; 89:2281-92. Doi: 10.3382/ps.2010.
264. Strijbos C, Schluck M, Bisschop J., Bui T, de Jong I, van Leeuwen M, von Tottleben M, et al. Consumer awareness and credibility factors of health claims on innovative meat products in a cross-sectional population study in the Netherlands. Elsevier. 2016; 54:13-22. WOS:000382592300002
265. Suresh A. Consumers' attitude towards meat consumption in India: insights from a survey in two metropolitan cities. Livestock Research for Rural Development. 2016; 28(3):
266. Szendro K. Consumer Perceptions, Concerns, and Purchasing Practices of Rabbit Meat in Hungary. Journal of Food Products Marketing. 2016; 22:683-693. WOS:000382768100004
267. Thorslund CA, Aaslyng MD, Lassen J. Perceived importance and responsibility for market-driven pig welfare: Literature review. Meat Science. 2017; 125: 37-45.
268. Tian Q,Hilton D, Becker M. Confronting the meat paradox in different cultural contexts: Reactions among Chinese and French participants. Appetite. 2016; 96:187-94. DOI:10.1016/j.appet.2015.09.009. Epub 2015 Sep 12.
269. Trocchia PJ, Janda S. A cluster analytic approach for consumer segmentation using the vegetarian/meatarian distinction. Journal of Food Products Marketing. 2003; 9:11-23. FSTA:2004-10-Ag1681
270. Tsegay T. Consumer perceptions and preferences of meat types in Harar and Haramaya towns, Ethiopia. Journal of Microbiology, Biotechnology and Food Sciences. 2012; 2(3):959-969.
271. Urland GR, Ito TA. Have your cake and hate it, too: Ambivalent food attitudes are associated with dietary restraint. Basic and Applied Social Psychology. 1010; 27:353-360. WOS:000233707700008
272. Van Wezemael L, Verbeke W, de Barcellos MD, Scholderer J, Perez-Cueto F. Consumer perceptions of beef healthiness: results from a qualitative study in four European countries. BMC Public Health. 2010; 10:342.
273. Van Zyl MK, Steyn NP, Marais ML. Characteristics and factors infuencing fast food intake of young adult consumers in Johannesburg, South Africa. Medpharm Publications (Pty) Ltd (PO Box 14804, Lyttelton, Gauteng 0157, South Africa). 2010; 23:124-130.
274. Vanhonacker F, Lengard V, Hersleth M, Verbeke W. Profiling European traditional food consumers. British Food Journal. 2010; 112(8):871-886

275. Vanhonacker F, Van Loo WJ, Gellynck X, Verbeke W. Flemish consumer attitudes towards more sustainable food choices. Appetite. 2013; 62:7-16
276. Vanhonacker F, Verbeke W. Buying higher welfare poultry products? Profiling Flemish consumers who do and do not. Poultry Science. 2009; 88:2702-11. doi: 10.3382/ps.2009.
277. Vartanian LR. Impression management and food intake. Current directions in research. Appetite. 2015; 86:74-80. DOI: 10.1016/j.appet.2014.08.021. Epub 2014 Aug 19.
278. Velde HT, Aarts N, Van Woerkum C. Dealing with ambivalence: Farmers' and consumers' perceptions of animal welfare in livestock breeding. Journal of Agricultural and Environmental Ethics. 2002; 15:203-219. WOS:000176156900005
279. Viana MM, Silva VLD, Trindade MA. Consumers' perception of beef burgers with different healthy attributes. LWT-Food Science and Technology. 2014; 59:1227-1232. WOS:000342475900006
280. Vida V. Consumer attitudes and preferences about the pork meat in Hungary. Applied Studies in Agribusiness and Commerce. 2013; 7.
281. Vukasovic T. European meat market trends and consumer preference for poultry meat in buying decision making process. World's Poultry Science Journal. 2014; 70:289-301. WOS:000337728900005
282. Wang WC, Worsley A. Healthy eating norms and food consumption. European Journal of Clinical nutrition. 2014; 68:592-601. FSTA:2014-08-Aj12423
283. Wardy W, Mena B, Nongtaodum S, Prinyawiwatkul W. Exploring The Drivers Of Purchase Intent And Consumer Satisfaction Of Chicken Eggs Using Principal Component Analysis And The Kano Model. 2014; 29:463-473. WOS:000346054500008
284. Wilson RP, Freeman A, Kazda MJ, Andrews TC, Berry L, Vaeth PAC, Victor RG. Lay beliefs about high blood pressure in a low- to middle-income urban African-American community: An opportunity for improving hypertension control. Elsevier Inc. 2002; 112:26-30.
285. Worsley A, Skrzypiec G. Do attitudes predict red meat consumption among young people?. Ecology of Food and Nutrition. 1998; 37:163-195. WOS:000077165900004
286. Yercan M, Uzmay A, Doğan ZA. Factors affecting consumer preferences between red meat and chicken meat; A case of Izmir. Faculty of Agriculture, Ege University.2014; 121-124.
287. Young-Mee Lee, Jung-Hyum, Kim, Min-june Lee. Mothers’ perceptions of children’s food behaviors: use of focus group interview study. Nutrition Research and Practice. 2008; 2:259-268.
288. Zanoli R, Scarpa R, Napolitano F, Piasentier E, Naspetti S, Bruschi V. Organic label as an identifier of environmentally related quality: A consumer choice experiment on beef in Italy. Renewable Agriculture and Food Systems. 2011; 28:70-79. WOS:000314928700007
289. Zhang X. Shanghai consumer studies: with attention to livestock, dairy and horticulture products. Landbouw-Economisch Instituut (LEI). 2001; 44.
290. Zheng Q, Wang HH. Chinese preferences for sustainable attributes for food away from home: A rank-ordered model. China Economic. 2016; 41:148-158. WOS:000389966300011
291. Akrofi, M.M.; Antwi, S.H.; Gumbo, J.R. Students in Climate Action: A Study of Some Influential Factors and Implications of Knowledge Gaps in Africa. Environments 2019, 6, 12.

292. Aschemann-Witzel, J., Ares, G., Thøgersen, J., & Monteleone, E. (2019). A sense of sustainability? – How sensory consumer science can contribute to sustainable development of the food sector. Trends in Food Science & Technology.
293. Bert F, Scaioli G, Tolomeo M, Lo Moro G, Gualano MR, Siliquini R. Knowledge, attitudes and eating habits red and processed meat among gym users: a cross-sectional survey. Perspect Public Health. 2019:1757913919883908.
294. Bir C, Davis M, Widmar N, Zuelly S, Erasmus M. Perceptions of Animal Welfare With a Special Focus on Turkeys. Front Vet Sci. 2019; 6:413.
295. Bruno, M., Thomsen, M., Pulselli, F.M. et al. The carbon footprint of Danish diets. Climatic Change 156, 489–507 (2019).
296. Chong FS, Farmer LJ, Hagan TDJ, Speers JS, Sanderson DW, Devlin DJ, et al. Regional, socioeconomic and behavioural- impacts on consumer acceptability of beef in Northern Ireland, Republic of Ireland and Great Britain. Meat Sci. 2019; 154:86-95.
297. Feinberg M, Kovacheff C, Teper R, Inbar Y. Understanding the process of moralization: How eating meat becomes a moral issue. J Pers Soc Psychol. 2019;117(1):50-72.
298. Kuase A, Bruine de Bruin W, Millward-Hopkins J, Olsson H. Public perceptions of how to reduce carbon footprints of consumer food choices. Environ. Res. Lett. 1a; 2019: 114005.
299. Krispenz, A.; Bertrams, A. Correlates of the Intention to Reduce Meat Consumption. Sustainability 2020, 12, 4774. https://doi.org/10.3390/su12114774
300. Mertens A, von Krause M, Meyerhofer S, Aziz C, Baumann F, Denk A, et al. Valuing humans over animals - Gender differences in meat-eating behavior and the role of the Dark Triad. Appetite. 2020;146:104516
301. Tarrega A, Rizo A, Murciano A, Laguna L, Fiszman S. Are mixed meat and vegetable protein products good alternatives for reducing meat consumption? A case study with burgers. Current Research in Food Science. 2020;3:30-40.
302. Willits-Smith A, Aranda R, Heller MC, Rose D. Addressing the carbon footprint, healthfulness, and costs of self-selected diets in the USA: a population-based cross-sectional study. Lancet Planet Health. 2020 Mar;4(3):e98-e106. doi: 10.1016/S2542-5196(20)30055-3. PMID: 32220679; PMCID: PMC7232940.
303. Jing Shi, Vivianne H.M. Visschers, Noëmi Bumann, Michael Siegrist.Consumers' climate-impact estimations of different food products. Journal of Cleaner Production. 2016
304. Kurz, V. (2018). Nudging to reduce meat consumption: Immediate and persistent effects of an intervention at a university restaurant. Journal of Environmental Economics and Management, 90, 317–341.
305. Harper, Gemma C. and Spencer Henson. "Consumer Concerns about Animal Welfare and the Impact on Food Choice." (2005).
306. Kang HK, Park KT, Jeon JJ et al. A Survey of Korean Consumers' Awareness on Animal Welfare of Laying Hens [2018]Hong, E.C., National Institute of Animal Science, Rural Development Administration, Pyeongchang, Republic of Korea.
307. Harray, Amelia J.; Meng, Xingqiong; Kerr, Deborah A.; Pollard, Christina M. (2018). Healthy and sustainable diets: Community concern about the effect of the future food environments and support for government regulating sustainable food supplies in Western Australia. Appetite, (), S0195666317313764–. doi:10.1016/j.appet.2018.01.009

308. Trevena J, Reeder A. Perceptions of New Zealand adults about reducing their risk of getting cancer. New Zealand Medical Association (26 The Terrace, P.O. Box 156, Wellington, New Zealand). 2007; 120

309. Carroll JA, Capel EM, Gallegos D. Meat, Masculinity, and Health for the "Typical Aussie Bloke": A Social Constructivist Analysis of Class, Gender, and Consumption. Am J Mens Health. 2019;13(6):1557988319885561.

310. Wang F, Basso F. "Animals are friends, not food": Anthropomorphism leads to less favorable attitudes toward meat consumption by inducing feelings of anticipatory guilt. Appetite. 2019;138:153-73.

311. Plante C.N., Rosenfeld D.L., Plante M. & Reysen S., The role of social identity motivation in dietary attitudes and behaviors among vegetarians, Appetite (2019)

312. Unni Kjaernes, Randi Lavik and Ingrid Kjoerstad. Animal friendliness and food consumption practices. Preliminary results from population surveys in 7 countries.

313. Rice M, Hemsworth LM, Hemsworth PH, Coleman GJ. The Impact of a Negative Media Event on Public Attitudes Towards Animal Welfare in the Red Meat Industry. Animals (Basel). 2020;10(4).

314. De Backer C., Erreygers S., De Cort C., Vandermoere F., Dhoest A., Vrinten J. & Van Bauwel S., Meat and masculinities. Can differences in masculinity predict meat consumption, intentions to reduce meat and attitudes towards vegetarians?, Appetite(2020).

315. Lea, Emma & Worsley, Anthony & Crawford, David. (2006). Australian Adult Consumers' Beliefs About Plant Foods: A Qualitative Study. Health education & behavior : the official publication of the Society for Public Health Education. 32. 795-808.

316. SM Shab, Choi SH, Chung WH. Consumers' Perceptions and Valuation of an Organic Chicken in Malaw [2018]

317. Kristine M. Grimsrud, Henrik Lindhjem, Ingvild Vestre Sem & Knut Einar Rosendahl (2020) Public acceptance and willingness to pay cost-effective taxes on red meat and city traffic in Norway, Journal of Environmental Economics and Policy, 9:3, 251-268

318. De Barcellos MD, Krystallis A, Saab MSD, Kugler JO, Grunert KG. Investigating the gap between citizens' sustainability attitudes and food purchasing behaviour: empirical evidence from Brazilian pork consumers. Journal on Chain and Network Science. 2011; 35:391-402. WOS:000292426800003

319. Izmirli AYS. A Survey on Animal Welfare Attitudes of Veterinary Surgeries, Veterinary Students, Animal Owners and Society in Turkey. Kafkas Univ Vet Fak Derg. 2010; 16(6):981-985.

320. James S. The chill chain ''from carcass to consumer''. Meat Science. 1996; 43:203-216. WOS:A1996VG83400017

321. Jezewska-Zychowicz M. Stereotypical thinking about food in the context of promoting weight gain. Roczn. Pzh. 58(2):437-444. FSTA:2008-01-Aj0383

322. Toma L, McVittie A ,Hubbard C, Stott AW. A Structural Equation Model of the Factors Influencing British Consumers' Behaviour toward Animal Welfare. Journal of Food Products Marketing. 2011; 17:2-3, DOI: 10.1080/10454446.2011.548748.

323. Zingg A, Siegrist M. People's willingness to eat meat from animals vaccinated against epidemics. Food Policy. 2012; 37:226-231.

324. Palomo-Vélez, Gonzalo; Tybur, Joshua M.; van Vugt, Mark (2018). Unsustainable, unhealthy, or disgusting?Comparing different persuasive messages against meat consumption. Journal of Environmental Psychology, (), S0272494418300446–.
325. " Pavlovski Z, Aleksić S, Cmiljanić R, Škrbić Z, Lukić M. Investigation of the consumer attitude towards beef as food stuff. Institute for Animal Husbandry. 2003; 19:19-24."
326. Bakker D, Dagevos H. Meat lovers, consumers of less meat and meat avoiders: sustainable protein consumption in a carnivorous food culture. Landbouw-Economisch Instituut (LEI). 2010; 207.
327. Consumer attitudes towards beef and beef technologies. 2011; 76:85-8.
328. Glitsch. Consumers' attitudes towards food: the case of meat in Western European countries. Wissenschaftsverlag Vauk Kiel KG. 2001; 231-244.
329. K. Glitsch. Consumer requirements for fresh meat: results of the survey. Wissenschaftsverlag Vauk Kiel KG. 2000; 113-155
330. Kayser M, Spiller A. The image of the different types of meat from a consumer perspective. Austrian Society of Agricultural Economics. 2011; 21:23-31.
331. Lima Filho DO, Sproesser RL, Tredezini CAO, Moretti L. Which drives poultry meat consumers: health or price?. Instituto de Economia Agrícola. 2005; 35:7-18.
332. MacKenzie M, Shanahan L. Attitudes to reducing meat consumption in urban vs. rural populations. Asociacion Espanola de Dietistas-Nutricionistas. 2016; 20:567.
333. Nesse K. [Differences in eating behavior of men and women. Fish for the lady, meat for the gentleman?].original title: Unterschiede im Essverhalten von Männern und Frauen. MMW Fortschritte der Medizin. 2008; 150:18.
334. Süllodouble acutens G, Solymosi V. Consumer habit of the meat industrial products in Hungary Part II. Consumer preferences. Hungarian Scientific Society for Food Industry. 2008; 62:251-252.
335. Boyle E. Consumer support for healthy, humane and sustainable food. World Society for the Protection of Animals. 2012; 161-163.
336. Kutluk T, Yalcin S. Cancer beliefs and behaviors survey in Turkey. Blackwell Publishing Ltd. 2014; 10:34.
337. Tomlinson C, Lycett D. A qualitative study exploring the role of religion on diet choices in Seventh-day Adventist University Students. 2016; 29:59-60. FSTA:2016-10-Aj9095
338. Carlucci DN, Giuseppe N, De Devitiis B, Viscecchia R, Bimbo F, Nardone G. Consumer purchasing behaviour towards fish and seafood products. Patterns and insights from a sample of international studies. Appetite. 2015; 84:212-27.: 10.1016/j.appet.2014.10.008. Epub 2014 Oct 22.
339. Corrin T, Papadopoulos A. Understanding the attitudes and perceptions of vegetarian and plant-based diets to shape future health promotion programs. Appetite. 2017; 109:40-47. FSTA:2017-06-Aj5177
340. Hamilton M. Eating death. Food, Culture & Society. 2006; 9:155-177. FSTA:2006-11-Ag3155
341. Hartmann C, Siegrist M. Consumer perception and behaviour regarding sustainable protein consumption: A systematic review. Trends in Food Science & Technology. 2017; 61:11-25. WOS:000396959800002

342. Ingenbleek PTM, Immink VM. Consumer decision-making for animal-friendly products: synthesis and implications. Animal Welfare. 2011; 20:11-19. WOS:000287062900003
343. Izmirli S, Phillips CJC. Attitudes to animal welfare and rights throughout the world in the modern era: a review. Selcuk Universitesi Veteriner Fakultesi.2012; 28:65-68.
344. Joyce A, Dixon S, Comfort J, Hallett J. Reducing the environmental impact of dietary choice: Perspectives from a behavioural and social change approach. Hindawi Publishing Corporation. 2012; 978672. doi:10.1155/2012/978672.
345. Lyles JL, Calvo-Lorenzo MS. Bill E. Kunkle Interdisciplinary Beef Symposium: practical developments in managing animal welfare in beef cattle: what does the future hold?. American Society of Animal Science. 2014; 92:5334-5344. FSTA:2015-03-Sg0606
346. Napolitano F, Girolami A, Braghieri A. Consumer liking and willingness to pay for high welfare animal-based products. Food Science & Technology. 2010b; 21:537-543. WOS:000285035700002
347. Ruby MB. Vegetarianism. A blossoming field of study. Appetite. 2012; 58:141-150. FSTA:2012-04-Aj2188
348. Sumpter KC. Masculinity and Meat Consumption: An Analysis Through the Theoretical Lens of Hegemonic Masculinity and Alternative Masculinity Theories. Sociology Compass. 2015; 9:104-114. WOS:000365814800002
349. Timm SC. Moral Intuition or Moral Disengagement? Cognitive Science Weighs in on the Animal Ethics Debate. Springer Netherlands. Neuroethics. 2016; 9:225-234.
350. Walley K, Parrott P, Custance P, Meledo-Abraham P, Bourdin A. A review of UK consumers' purchasing patterns, perceptions and decision making factors for poultry meat. World's Poultry Science Journal. 2014; 70:493-501. WOS:000342509600005
351. Rothgerber H., Meat-related cognitive dissonance: A conceptual framework for understanding how meat eaters reduce negative arousal from eating animals, Appetite (2019).
352. Begue L, Treich N. Immediate and 15-Week Correlates of Individual Commitment to a "Green Monday" National Campaign Fostering Weekly Substitution of Meat and Fish by Other Nutrients. Nutrients. 2019;11(7).
353. Carfora V, Bertolotti M, Catellani P. Informational and emotional daily messages to reduce red and processed meat consumption. Appetite. 2019;141:104331.
354. Brunner, F., Kurz, V., Bryngelsson, D., & Hedenus, F. (2018). Carbon Label at a University Restaurant – Label Implementation and Evaluation. Ecological Economics, 146, 658–667.
355. Camilleri, A.R., Larrick, R.P., Hossain, S. et al. Consumers underestimate the emissions associated with food but are aided by labels. Nature Clim Change 9, 53–58 (2019).
356. Jay, J.A., D'Auria, R., Nordby, J.C. et al. Reduction of the carbon footprint of college freshman diets after a food-based environmental science course. Climatic Change 154, 547–564 (2019).
357. Vainio A., How consumers of meat-based and plant-based diets attend to scientific and commercial information sources: Eating motives, the need for cognition and ability to evaluate information, Appetite (2019), doi: https://doi.org/10.1016/j.appet.2019.03.017.

358. de Boer J; Carolien T. Hoogland; et al. (2007) Towards more sustainable food choices: Value priorities and motivational orientations. Food Quality and Preference 18(7), 0–996.
359. Mooney KM, Walbourn L. (2001) When college students reject food: not just a matter of taste. Appetite 36(1):41-50.

Supplement Table 6. Risk-of-Bias Assessments for Quantitative Studies

Study ID*	Selection of participants. Was an appropriate study sample selected from the sampling frame?	Missing data. Was the response rate sufficiently high to minimize the risk of bias?	Measurement instrument. Did the researchers pilot the measurement techniques on a subset of the target population?	Was the instrument validated?	Was the instrument reliable?	Overall risk of bias
**Reasons for eating and/or buying meat**						
Apostolidis 2019	Yes	Can't tell	Yes	Can't tell	No	Moderate
Eldesouky 2020	Yes	Can't tell	Yes	No	No	High
Frewer2005	Yes	Yes	Yes	Yes	Yes	Low
Grunert 2018	Yes	Can't tell	No	No	Yes	High
Koistinen 2013	Yes	No	No	No	No	High
McCarthy 2003	Yes	Can't tell	No	Yes	Yes	Low
McCarthy 2004	Yes	Can't tell	No	Yes	Yes	Low
Peneau 2017	Yes	No	No	Yes	No	Moderate
**Reasons for avoiding meat**						
Cordts 2014	Yes	Yes	No	No	No	High
Crnic 2013	Can't tell	Can't tell	No	No	No	High
De Backer 2014	Can't tell	Yes	No	Yes	No	High
de Gavelle 2019	Yes	No	No	Can't tell	Yes	High
Dyett 2013	Yes	Yes	Yes	No	No	High
Hagmann 2019	Yes	No	No	Yes	Yes	High
Haverstock 2012	No	can't tell	Yes	No	No	High
Herzog2009	No	Can't tell	No	Yes	Yes	High
Hoffman 2013	Yes	Yes	No	No	Yes	High
Hopwood 2020	Yes		No	Yes	Yes	Low
Izmirli2011	Can't tell	Can't tell	Yes	Yes	No	Moderate
Kayser 2013	Can't tell	Yes	Yes	Yes	Yes	Low
Lea 2003	No	Yes	Yes	Yes	Yes	Low
Lea 2004	Yes	Yes	Yes	yes	Yes	Low
Lentz 2018	Yes	Yes	No	Other	Yes	Low
Lindeman 2001 (study 1)	No	Can't tell	No	yes	yes	Moderate
Lindeman 2001 (study 2)	No	Can't tell	No	yes	No	Moderate
Mullee 2017	Yes	No	No	No	No	High
Neff 2018	Yes	Yes	Yes	No	No	High
Peneau 2017	Yes	No	No	Yes	Yes	Moderate
Philips 2011	No	Can't tell	Yes	yes	No	Moderate

Ploll 2019	Yes	Can't tell	Yes	Can't tell	Yes	High
Povey 2001	No	No	No	Can't tell	Yes	High
Pribis 2010	No	Can't tell	No	No	No	High
Ruby 2013 (Study 1)	Yes	Can't tell	No	Yes	Yes	Low
Schösler 2015	Yes	Can't tell	No	No	No	High
Siegrist 2019	No	No	No	Yes	Yes	Moderate
Spencer 2007	No	Yes	No	Yes	No	Moderate
Verain 2015	Yes	Yes	No	Yes	yes	Low
Austgulen 2018	Yes	Can't tell	No	No	No	High
**Willingness to Change Meat Consumption**						
Asvatourian 2018	Yes	No	No	Yes	No	Moderate
Bryant 2019	Yes	Can't tell	No	No	No	High
Clonan 2015	Yes	No	Yes	Yes	Yes	Low
Cordts 2014	Yes	Yes	No	No	No	High
de Boer 2013	Yes	Yes	No	Yes	Yes	Low
de Boer 2016	Can't tell	Can't tell	No	Yes	Can't tell	Moderate
de Boer 2018	Can't tell	Yes	No	No	Yes	High
De Groeve 2017	No	No	No	No	Yes	High
Ginn 2019 (study 1)	No	Can't tell	No	No	No	High
Ginn 2019 (study 2)	No	Can't tell	No	No	No	High
Hunter 2016	No	No	No	Yes	Yes	Moderate
Latvala 2012	Yes	No	No	No	No	High
Lea 2003	No	Yes	No	No	No	High
Lea 2008	No	No	Can't tell	No	Yes	High
Lentz 2018	Can't tell	Yes	No	Other	Yes	Low
Mäkiniemi 2014	No	Can't tell	Can´t tell	No	No	High
Malek 2019	Yes	Yes	No	No	Yes	High
McCarthy 2004	Yes	Can't tell	No	Yes	Yes	Low
Neff 2018	Yes	Yes	Yes	No	No	High
Pohjolainen 2016	Yes	No	No	Can't tell	No	High
Schösler 2015	Yes	Can't tell	No	No	No	High
Siegrist 2019	No	No	No	Yes	Yes	Moderate
Tobler 2011	Can't tell	No	No	No	Yes	High
Truelove 2012	Can't tell	Can't tell	No	No	Yes	High
Vanhonacker 2013	No	Can't tell	No	Yes	Yes	Moderate
Verain 2015	Yes	Yes	No	Yes	yes	Low

Siegrist 2015	can't tell	No	No	No	No	High
**Willingness to pay more for environmentally friendly meat**						
Akaichi2020	Can't tell	Can't tell	Yes	No	No	High
Eldesouky 2020	Yes	Can't tell	Yes	No	No	High

Supplement Table 7. Methodological Limitations Assessments for Qualitative Studies

**Study ID***	**Was there a clear statement of the aims of the research?**	**Is a qualitative methodology appropriate?**	**Was the research design appropriate to address the aims of the research?**	**Was the recruitment strategy appropriate to the aims of the research?**	**Was the data collected in a way that addressed the research issue?**	**Has the relationship between researcher and participants been adequately considered?**	**Have ethical issues been taken into consideration?**	**Was the data analysis sufficiently rigorous?**	**Is there a clear statement of findings?**	**How valuable is the research?**	**Overall methodological limitations**
**Reasons for eating and/or buying meat**											
Mceachern 2002	Yes	Yes	Yes	Yes	Yes	No	No	Can't tell	Yes	Yes	Moderate methodological limitations
Fox 2008	Yes	Yes	Yes	Yes	Yes	Yes	Can't tell	Yes	yes	yes	No or minor methodological limitations
Happer 2019	Yes	Yes	Yes	Yes	Yes	No	No	No	Yes	Yes	Serious methodological limitations
Myceck 2018	Yes	Yes	Can't tell	yes	yes	yes	No	yes	yes	yes	No or minor methodological limitations
Mylan 2018	Yes	Yes	Yes	yes	yes	No	Can't tell	No	Yes	Yes	Moderate methodological limitations
Boyle 2011	yes	yes	yes	yes	yes	Can't tell	yes	yes	yes	yes	No or minor methodological limitations
Guerin 2014	yes	yes	yes	yes	yes	yes	yes	Can't tell	yes	yes	No or minor methodological limitations

Lea 2005	yes	yes	yes	yes	yes	No	yes	yes	yes	yes	No or minor methodological limitations
**Willingness to Change Meat Consumption**											
Happer 2019	Yes	Yes	Yes	Yes	Yes	No	No	No	Yes	Yes	Serious methodological limitations
Macdiarmid 2016	Yes	Yes	Yes	Yes	Yes	Yes	Yes	Yes	Yes	Yes	No or minor methodological limitations
Mceachern 2002	Yes	Yes	Yes	Yes	Yes	No	No	Can't tell	Yes	Yes	Moderate methodological limitations
Graça 2014	Yes	Yes	Yes	yes	yes	No	yes	yes	Yes	Yes	No or minor methodological limitations
Hoek 2016	yes	yes	yes	yes	yes	Can't tell	yes	yes	Yes	Yes	No or minor methodological limitations
Spendrup 2017	yes	yes	yes	Can't tell	yes	No	No	yes	Yes	Yes	Moderate methodological concerns
Lea 2005	yes	yes	yes	yes	yes	No	yes	yes	Yes	Yes	No or minor methodological limitations

Supplement Table 8. Critical appraisal for Mixed-Methods studies

Study ID	Is there an adequate rationale for using a mixed methods design to address the research question?	Are the different components of the study effectively integrated to answer the research question?	Are the outputs of the integration of qualitative and quantitative components adequately interpreted?	Are divergences and inconsistencies between quantitative and qualitative results adequately addressed?	Do the different components of the study adhere to the quality criteria of each tradition of the methods involved?	Overall assessment
Austgulen 2018[1]	Yes	Yes	Yes	Yes	Yes	Clear and detailed information and justification to use the mixed-methods approach are provided.
Scott 2019[2]	No	No	No	No	No	No information is provided for using a mixed-methods approach, unclear how the quantitative evidence contributed to the findings.

[1] Contributes with quantitative evidence for the finding "**Reasons for avoiding meat"** and with qualitative evidence for the finding **"Willingness to change meat consumption".**

[2] Contributes with qualitative evidence for the finding **"Willingness to change meat consumption".**

Supplement Table 9. Evidence Profile for "Reasons for eating and/or buying meat"

Review finding	N° of studies (participants)	Methodological limitations	Coherence	Relevance	Adequacy of data	Confidence of evidence
**Reasons for eating and/or buying meat - Integrated evidence**						
**Consumers chose meat with a lower footprint, when provided with carbon footprint information of meat production** (25, 26, 29). However, other characteristics such as type of meat (25), fat content (25,29) and price were considered more important (29<br><br>**The environment (for example, carbon footprint information on the label) was not considered a significant aspect when buying/consuming meat** (27, 28; 30-32); other aspects such as: nutritional values (28,32), freshness of the meat (27), food safety (27, 28, 30,31), eating enjoyment/taste (27,31), and animal welfare (28, 31) were considered more important.<br><br>**Consumers bought meat products based on tangible aspects such as colours and appearance rather than intangible characteristics such as environmental aspects of production;** only some participants bought environmentally friendly meat products, the main barriers were the higher price of these products and the unwillingness to change their diet (92).	9 (N=28,953)[1]	Moderate concerns[2]	No concerns	Serious concerns[3]	No concerns	LOW ⊕⊕◯◯

Abbreviations: QUAL=Qualitative, QUANT= Quantitative.

[1] **QUANT:** Eight studies (N=28,923) conducted in Finland, France, Germany, Ireland, Netherlands, Poland, Spain, and United Kingdom. **QUAL:** One study (N=30) conducted in Scotland.
[2] **QUANT:** Three studies (38%) were at high risk of bias for lack of validation of the measurement instruments, 2 (25%) at moderate risk of bias, and 3 (37%) at low risk of bias. **QUAL:** Moderate methodological limitations due to lack of reporting of the investigator and participants' relationship, lack of reporting of ethical issues, and limited information on the analysis process.
[3] **QUANT:** Serious concerns because five studies (63%) did not inform participants about the environmental impact of meat consumption. **QUAL:** Serious concerns because one study (100%) did not inform participants about the environmental impact of meat consumption.

Supplement Table 10. Evidence Profile for "Reasons for avoiding meat"

Review finding	N° of studies (participants)	Methodological limitations	Coherence	Relevance	Adequacy of data	Confidence of evidence
**Reasons for avoiding meat – Integrated evidence**						
**For vegetarians and low meat consumers/meat reducers, the reasons for adopting a vegetarian diet or limiting their meat intake varied** (35, 37,39-42, 43,44, 45, 46, 47, 48, 49, 50, 53,54, 55,56,57,59,96). For many people, environmental concerns were among the most important reasons for avoiding meat consumption (35, 43, 44, 46, 48, 49, 50, 53, 55, 56), whereas for others, environmental concerns were not considered one of the main reasons for avoiding meat (35, 37, 39-42, 45, 47, 54, 57, 59,96).<br><br>**Environmental concerns were considered a contributory factor rather than the primary driver for avoiding meat** (87,88). However, environmental impact of meat production was mentioned as one reason for avoiding meat intake by some participants (84), along with other reasons, for example perceived health (90). Other reasons such as: animal welfare (84,85); health concerns (85,94); self-fulfilment and taste or aesthetics (84) were considered among the main reasons for avoiding meat.<br><br>**Women were more likely to avoid meat or eating smaller portions of meat for environmental reasons** (33, 39, 50, 60), except for one study where men were more likely to report environmental concerns as a reason for avoiding meat (52).<br><br>**The younger population was more likely to agree that a vegetarian diet leads to environmental benefits** (45, 55).<br><br>**People's meat consumption behaviour influenced their motivations for avoiding meat intake** (34, 47, 38, 87). The stricter the diet in terms of avoiding meat consumption and animal products, the more important environmental concerns were reasons for avoiding meat (**Error! Reference source not found.**, **Error! Reference source not found.**, **Error! Reference source not found.**). Similarly, one study reported that all vegans found the environment an important issue for meat consumption, while only a minority of omnivores mentioned it (87).	37 (N=63,208)[1]	Minor concerns[2]	Minor concerns[3]	Serious concerns[4]	No concerns	LOW ⊕⊕◯◯

__Abbreviations:__ MM=Mixed-methods, QUAL=Qualitative, QUANT= Quantitative.

[1] **QUANT:** Twenty-nine studies (N=61,219) conducted in Australia, Austria, Belgium, Czech Republic, Finland, France, Ireland Germany, Macedonia, Netherlands, New Zeeland, Norway, Slovenia, Serbia Spain, Sweden, Switzerland, United Kingdom, and United Stated of America. **QUAL:** Seven studies (N=457) conducted in Australia, Austria, Canada, China, Brazil, United Kingdom, United States of America.**MM**: One study (1,532) conducted in The Netherlands.

[2] **QUANT:** Sixteen studies (54%) were at high risk of bias for lack of validation of the measurement instruments, 7 (23%) were at moderate risk of bias, and 7 (23%) were at low risk of bias. **QUAL:** Minor methodological limitations to lack of reporting of the investigator and participants' relationship, lack or limited information on ethical issues, and lack or limited information on the analysis process. **MM:** No concerns, clear and detailed information and justification to use the mixed-methods approach are provided.

[3] Reasons for avoiding meat intake changed and varied across studies, no clear reasons for this variability were identified.

[4] **QUANT:** Serious concerns because 29 studies (100%) did not inform participants about the environmental of meat consumption. **MM** study contributing to QUANT evidence did not provide information to participants. **QUAL:** Serious concerns because 6 studies (86%) did not inform participants about the environmental impact of meat consumption.

Review finding	N° of studies (participants)	Methodological limitations	Coherence	Relevance	Adequacy of data	Confidence of evidence
**Willingness to change meat consumption – Integrated evidence**						
**Most of the omnivores were reluctant to reduce meat consumption in the future** (60,61,67,64,65,71,72,77,80,81,82,83, 86, 89, 91, 96), even when informed on the environmental impact of meat consumption (33,67,70).<br><br>Similarly, **when provided with an information sheet** about the impact of food production on climate change, **most of the participants showed low awareness of the association between climate change and meat consumption, and some participants reported considering reducing their meat consumption or had already reduce their intake in the past.** However, environmental concerns tended to be a contributory factor rather than the primary driver; other aspects were considered more important for the environment rather than reducing meat consumption (88).<br><br>**Most of omnivores were willing to adopt other strategies to reduce the climate impact rather than reducing meat intake**: eating more organic food (69, 77, 83), driving less (81), eating local foods (77,83); using alternate transportation, recycling (81), using eco-friendly products (81), reporting the ecological impact on the food's labels (67). On the contrary, three studies reported that **most of the participants, when presented with different sustainable food behaviours they could choose from, they were willing to reduce their meat intake in terms of quantity** (60, 66,82) rather than eating plant-based meat substitutes and proteins from insects (85) or buying specific meat such as organic meat (60) or replace most of the meat by vegetables (66). **Omnivores considered meat consumption to have a trivial effect on the environment and believed that other behaviours were more effective** (91). Food packaging, food waste, transportation of food, and production and processing of food in relation to the environmental impact of food were considered more important (91).<br><br>**Women** perceived higher environmental benefit of eating less meat than men and **were more willing to reduce meat intake** (60,62-67,70, 71,75,78, 80,81,83). **Young women were most incline to change their meat consumption** (96)<br><br>**Frequent meat consumers were less positive towards a reduction of meat** (65-69, 74, 80), whereas those with higher concerns for environmental problems were much more likely to intend to stop eating meat (64,67,68,69,80,81). On the contrary, one study found that gender,	38 (N=57,148)[1]	Minor concerns[2]	Minor concerns[3]	Serious concerns[4]	No concerns	LOW ⊕⊕◯◯

as well as age, meat consumption behaviour (high vs. low intake) and socio-economic status had no impact on peoples' belief that eating less meat would help reducing climate change (63).						

Abbreviations: MM=Mixed-methods, QUAL=Qualitative, QUANT= Quantitative.

[1] **QUANT:** Twenty-seven (N=56,555) conducted in Australia, Austria, Belgium, Bulgaria, Cyprus, Czech Republic, Denmark, Estonia, Finland, France, Germany, Greece, Hungary, Ireland, Italy, Latvia, Lithuania, Luxembourg, Malta, Netherlands, New Zeeland, Poland, Portugal, Romania, Slovakia, Slovenia, Southwest Scotland, Spain, Sweden, Switzerland, United Kingdom, United Sates of America. **QUAL:** Seven studies (N=527) conducted in Australia, Brazil, China, Norway, Portugal, Scotland, Sweden, United Kingdom, and United States of America. **MM:** Two studies (N=66) conducted in Spain and Norway.
[2] **QUANT:** Eighteen studies (60%) were at high risk of bias for lack of validation of the measurement instruments, 6 (20%) at moderate risk of bias and 6 (20%) at low risk of bias.
QUAL: Minor methodological limitations due to lack of reporting of the investigator and participants' relationship and lack of information on ethical issues. **MM:** One study was there were no concerns, clear and detailed information and justification to use the mixed-methods approach are provided. For the second study, no information is provided for using a mixed-methods approach, unclear how the quantitative evidence contributed to the findings.
[3] **QUANT:** Minor concerns because three studies reported contradictory data regarding the willingness to adopt other strategies to reduce climate impact, representing 45% of the overall population.
[4] **QUANT:** Serious concerns because 27 studies (100%) did not inform participants about the environmental impact of meat consumption. **QUAL:** Serious concerns because seven studies (87%) did not inform participants about the impact of meat consumption on the environment. Two **MM** studies contributing to qualitative evidence did not provide information to participants.

Supplement Table 12. Evidence Profile for "Willingness to pay more for environmentally friendly meat"

Review finding	N° of studies (participants)	Methodological limitations	Coherence	Relevance	Adequacy of data	Confidence of evidence
**Willingness to pay more for environmentally friendly meat- Quantitative evidence**						
**Most consumers** were willing to pay more for meat products if the product was produced with a significantly lower environmental impact (26, 79). Also, labels indicating that the beef mince had a low or moderate fat content (79), was organic meat produced locally and with animal welfare standards were significant for consumers (26).<br><br>**Women** and older people showed higher willingness to pay more for meat with minimal environmental impact (26,79).	2 (N=2,702)	No or minor concerns[2]	No concerns	Serious concerns [3]	No or minor concerns	MODERATE ⊕⊕◯◯

Abbreviations: QUANT= Quantitative.

[1] **QUANT:** Two studies 2 (N=2,702) conducted in United Kingdom and Spain.
[2] **QUANT:** All studies (100%) were at high risk of bias for lack of validation of the measurement instruments; however, findings were consistent across studies.
[3] **QUANT:** Serious concerns of relevance because all studies (100%) did not inform participants about the environmental impact of meat consumption.
